# Exploration and stabilization of Ras1 mating zone: A mechanism with positive and negative feedbacks

**DOI:** 10.1371/journal.pcbi.1006317

**Published:** 2018-07-20

**Authors:** Bita Khalili, Laura Merlini, Vincent Vincenzetti, Sophie G. Martin, Dimitrios Vavylonis

**Affiliations:** 1 Department of Physics, Lehigh University, Bethlehem, Pennsylvania, United States of America; 2 Department of Fundamental Microbiology, University of Lausanne, Lausanne, Switzerland; King’s College London, UNITED KINGDOM

## Abstract

In mating fission yeast cells, sensing and response to extracellular pheromone concentrations occurs through an exploratory Cdc42 patch that stochastically samples the cell cortex before stabilizing towards a mating partner. Active Ras1 (Ras1-GTP), an upstream regulator of Cdc42, and Gap1, the GTPase-activating protein for Ras1, localize at the patch. We developed a reaction-diffusion model of Ras1 patch appearance and disappearance with a positive feedback by a Guanine nucleotide Exchange Factor (GEF) and Gap1 inhibition. The model is based on new estimates of Ras1-GDP, Ras1-GTP and Gap1 diffusion coefficients and rates of cytoplasmic exchange studied by FRAP. The model reproduces exploratory patch behavior and lack of Ras1 patch in cells lacking Gap1. Transition to a stable patch can occur by change of Gap1 rates constants or local increase of the positive feedback rate constants. The model predicts that the patch size and number of patches depend on the strength of positive and negative feedbacks. Measurements of Ras1 patch size and number in cells overexpressing the Ras1 GEF or Gap1 are consistent with the model.

## Author summary

Unicellular fission yeasts mate by fusing with partners of the opposite mating type. Each pair member grows towards its selected partner that signals its presence through secreted pheromone. The process of partner selection occurs through an exploratory patch (containing activated signaling protein Cdc42 and upstream regulator Ras1) that assembles and disassembles on the cell cortex, stabilizing in regions of higher opposite pheromone concentration. We present a computational model of the molecular mechanisms driving the dynamical pattern of patch exploration and stabilization. The model is based on reaction and diffusion along the curved cell membrane, with diffusion coefficients measured experimentally. In the model, a positive Ras1 activation feedback loop generates a patch containing most of the activating protein (Ras1 GEF). The fast diffusing inhibitor Gap1 that is recruited locally from the cytoplasm spreads on the cell membrane, limiting patch size and causing its decay. Spontaneous reinitiation of Ras1 activation elsewhere on the cortex provides a mechanism for exploration. Transition of the system’s behavior to that of a single stable patch is possible upon simulated pheromone sensing. The computational model provides predictions for the number of patches and patch size dependence on parameters that we tested experimentally.

## Introduction

How cells sense a chemical concentration gradient and polarize towards regions of high or low concentrations is a fundamental biological question. Encoding of spatial information in chemoattractant gradients is essential for many processes, including the directional migration of leukocytes and neutrophils or the directional growth of yeast and neurons [1–4]. Cellular response of eukaryotic cells in response to extracellular cues generally involves establishing a cellular axis of polarity through accumulation of small GTPases, such as Ras and Cdc42, in cortical domains [5]. The establishment of such a self-organizing polarity zone is a process of pattern formation on the cell cortex.

**Fig 1:**
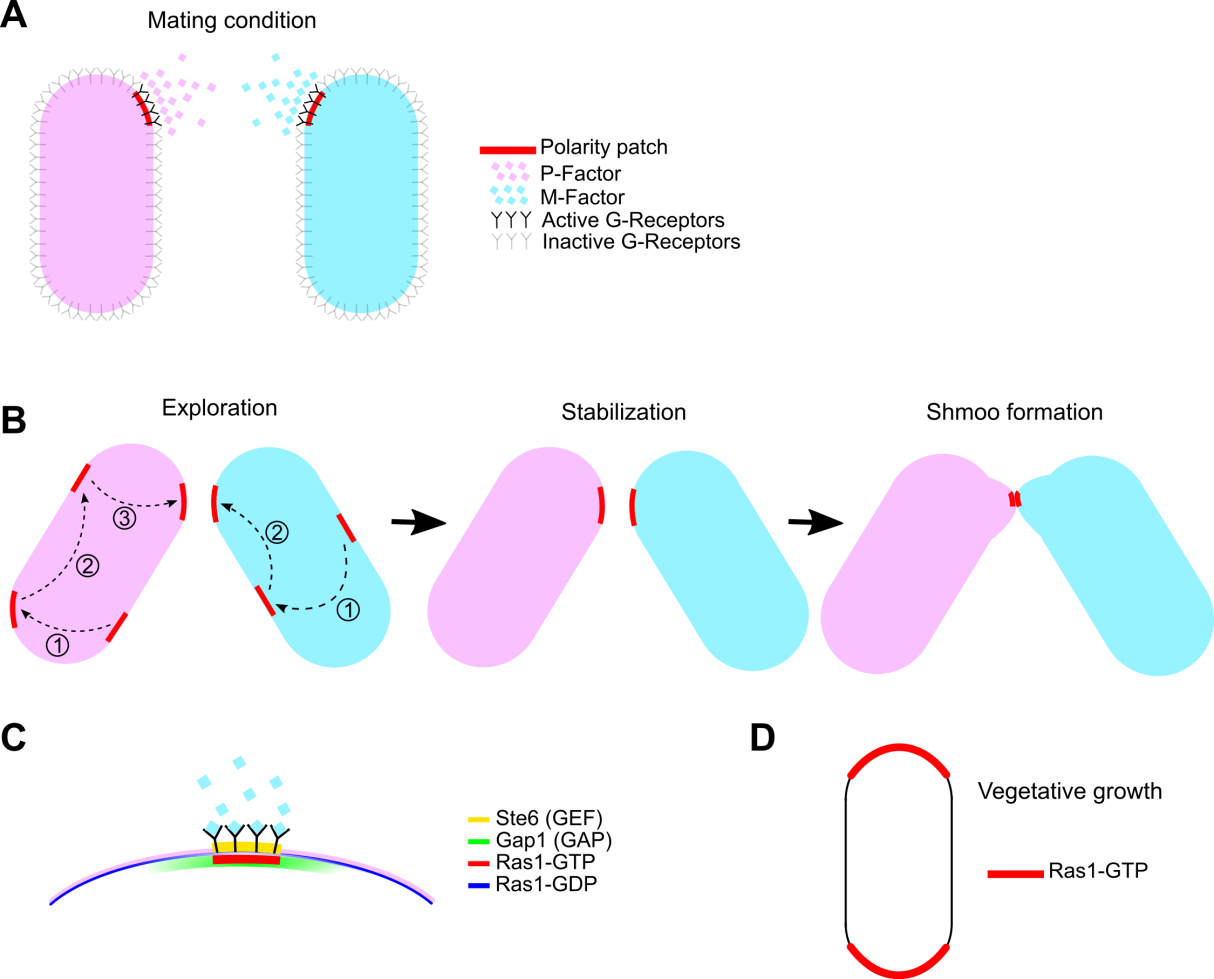
Schematic of fission yeast cells pheromone signaling and polarization. (A) When fission yeast cells undergo sexual differentiation, two opposite mating types P-and M-cells each secrete their own specific pheromones. Both the pheromone secretion and sensing happens locally from the polarity zones. (B) The polarity patch explores the cell periphery in discrete jumps. The dashed arrows show a sequence of patch appearance and disappearance. After few rounds of exploration it stabilizes close to the position with highest pheromone concentration from the partner. Then they grow a mating projection (shmoo) toward each other. Upon cell wall-cell wall contact the two cells fuse together. (C) The underlying signaling pathway of polarity patch formation. Upon binding of the pheromones to their cognate receptors on the surface of opposite mating type cells the MAPK pathway gets activated which in turn regulates the expression of Ste6, the GEF for Ras1. Ras1-GTP recruits its own GAP, Gap1, on the membrane. Gap1 localization is broader than Ras1-GTP. (D) Ras1-GTP localizes at the tips during vegetative growth (red) while Ras1-GDP extends across the sides.

As model eukaryotic cells, budding and fission yeasts have been used to study basic properties of chemical sensing mechanisms [6–8]. Under mating conditions, these organisms respond to the pheromone gradient secreted by the opposite mating type cells, in order to identify the closest potential partner, grow towards it by formation of a shmoo extension, and fuse with it [7]. Recent studies have shown that this process involves an initial assembly of a “patch” or “zone” that is established independently of the direction of the pheromone gradient (Fig 1A and 1B) [9–11]. Over time, the patch that contains activated Cdc42 and co-factors reorients and stabilizes towards the chosen pheromone-secreting partner and starts growing toward it via Cdc42-dependent recruitment of the cell growth machinery. The partner search strategy is different between the two yeasts. The budding yeast patch executes a biased diffusion along the cell cortex towards high pheromone concentration regions [10, 12, 13]. By contrast, fission yeast implements an exploratory search involving complete patch disappearance and re-appearance at a different cortical location, followed by patch stabilization (Fig 1B) [9]. It has been proposed that the fission yeast strategy helps the whole cell population explore space of possible mating configurations, thus optimizing population mating efficiency [11]. Furthermore, the localization of pheromone transporter and components of the signal transduction cascade suggest that the spatial gradient information is produced and sensed locally from the polarity zone [11].

Models of the mechanism of yeast pheromone gradient sensing have to explain both the ability of cells to organize a patch and the change of its location. Many theoretical studies have considered the process of Cdc42 patch formation for bud growth during the mitotic cell cycle. In budding yeast cells lacking Ras-like Rsr1, the bud forms randomly along the mother cell cortex through a process of spherical symmetry breaking [14]. Theoretical studies have suggested that a positive feedback in Cdc42 activation gives rise to a Turing-type pattern formation mechanism which breaks symmetry and establishes a single patch through amplification of a stochastic fluctuation at a random location [15, 16]. However, the relative contributions of Cdc42 activity regulators and actin to the positive feedback have been debated [12, 17–20]. To explain observed oscillations between competing patches in initial stages of bud site selection required implementing an additional negative feedback mechanism [21]. During budding yeast mating, experiments and mathematical models have suggested two different mechanisms for patch diffusion toward the pheromone gradient. McClure et al. suggested that perturbations caused by the localized delivery of vesicles near the patch site dilute polarity factors, an effect counteracted by polarized G proteins [13]. Hegemann et al. proposed that a secondary positive feedback between the actin-based receptor trafficking and Cdc42 allows for patch diffusion toward the gradient that stabilizes in the high concentrations of Cdc42-GTP [10].

Our aim in this work is to propose and test a model implementing a mechanism for fission yeast mating patch formation, exploration, and stabilization, a process that has not been explored theoretically. This study is motivated by recent findings emphasizing the role of Ras1, the only Ras GTPase homolog in fission yeast. Ras1 is an upstream regulator for Cdc42 and its activity is critical for both mating and polarity establishment during interphase, since *ras1*Δ cells are sterile and round [22]. During early stages of mating, active Ras1 (Ras1-GTP) localizes at the same exploratory sites as active Cdc42 [23]. The appearance and disappearance of the patch is an evidence for regulation through positive and negative feedback loops. Indeed, in *S*. *pombe*, Ras1 activation in mating conditions is promoted by the pheromone-induced Guanine nucleotide Exchange Factor (GEF) Ste6 [24]. It has been shown that Ste6 pheromone-induced expression is itself regulated by Ras1 activity, which in turn creates an additional positive feedback loop [25, 26]. Ras1 inactivation is promoted through interaction with its GTPase activating protein (GAP), Gap1. The deletion of Gap1 abolishes the Ras1 polarity patch [23], increases the lifetime of the Cdc42 patch during exploration and results in reduction of mating efficiency [11]. It was found that Gap1 is recruited to the active Ras1 patch, occupying a larger area on the membrane where it remains for a longer time compared to Ras1-GTP during patch disappearance [11] (Fig 1C). These observations are consistent with Gap1-dependent hydrolysis of Ras1-GTP forming a local negative feedback to temporally control the polarity patch.

In this study we developed a 3D reaction-diffusion model, which accounts for the fission yeast cell geometry, to study the dynamic regulation of Ras1 during fission yeast mating. To develop this model, we performed experiments to measure the kinetics of diffusion and membrane dissociation of active Ras1 (Ras1-GTP), inactive Ras1 (Ras1-GDP), and Gap1. Since many of the biochemical interactions of the Ras1 positive and negative feedbacks are not yet known we adopt a phenomenological approach, inspired by prior studies in budding yeast. We illustrate that by tuning the rate constants for the positive and negative feedback loops, the model can reproduce the appearance and disappearance of Ras1 patch at random locations. We investigate the switch to patch stabilization upon sensing of a high level of pheromone and show that the patch in our model can be stabilized at positions with a higher positive feedback rate constant, which might represent regions of higher pheromone concentration. The model predicts that the patch size can be regulated by positive and negative feedback. In simulations, increase in negative feedback resulted in narrower patches and increase in positive feedback resulted in formation of multiple patches. These results were tested and confirmed experimentally.

## Results

### Measurement of Ras1-GTP, Ras1-GDP and Gap1 diffusion coefficients

**Fig 2:**
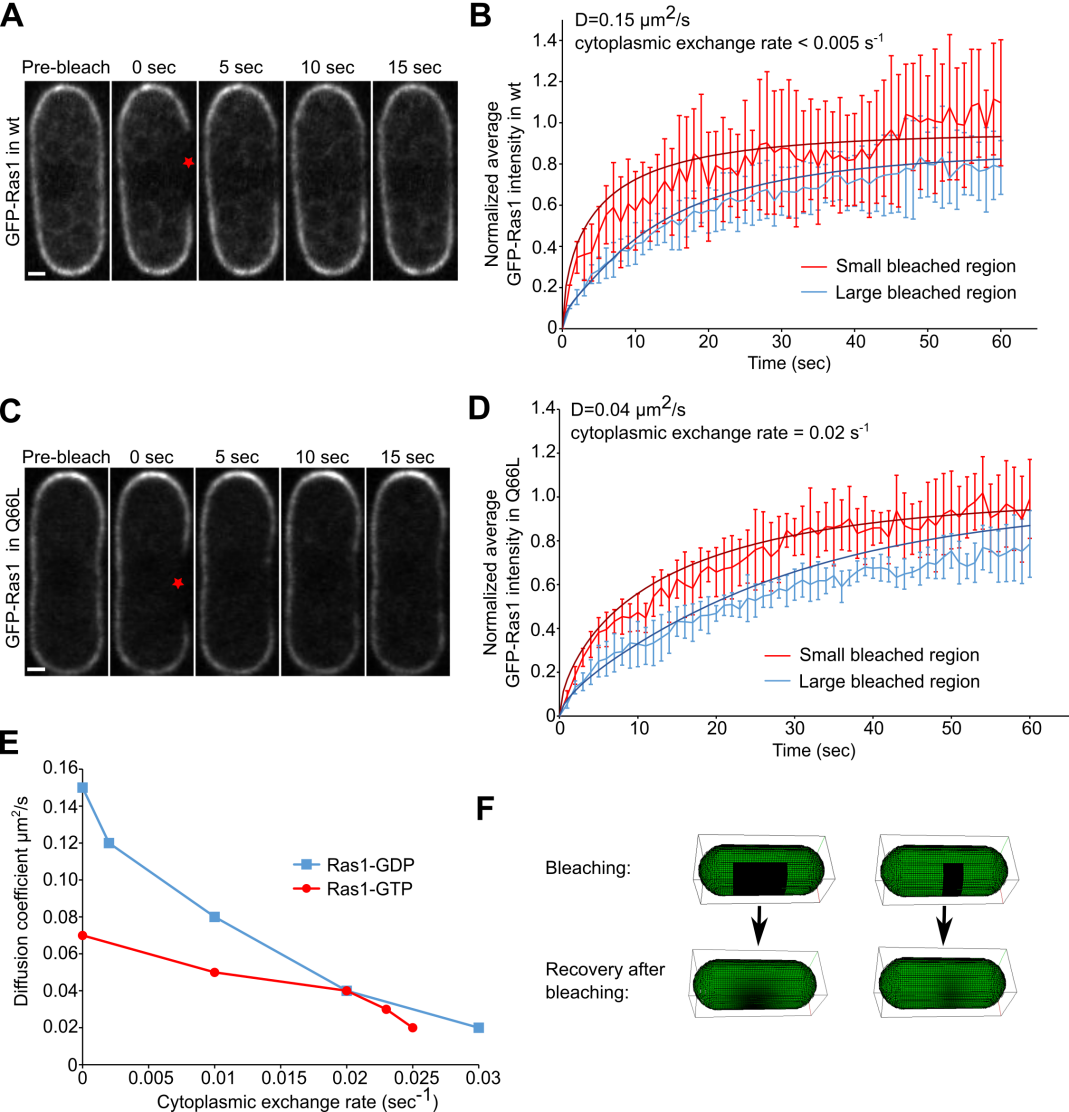
FRAP studies of Ras1 membrane diffusion. (A) Snapshots of FRAP of GFP-Ras1 at the sides of a WT cell (bleach region indicated by star). The scale bar is 1 μm. (B) Recovery of GFP-Ras1 at the sides of WT cells bleached over large (4.0 ± 0.2 μm) and small (1.5 ± 0.2 μm) regions, average of 5 cells. Measurements are made along the cell membrane, over the whole width of the bleached region. Continuous curves show fit by model with *D* = 0.15 ${\mu m}^{2}s^{-1}$ and no cytoplasmic exchange. (C) Snapshots of FRAP of GFP-Ras1 at the sides of *GFP-ras1*^*Q66L*^ cells. The scale bar is 1 μm. (D) Same as panel B for GFP-Ras1 at the sides of *ras1*^*Q66L*^ cells and fit with *D* = 0.04 ${\mu m}^{2}s^{-1}$, and uniform cytoplasmic exchange rate 0.02 $s^{-1}$. (E) Plot showing acceptable set of diffusion coefficients and cytoplasmic exchange rates that can be fitted to the FRAP data of panels A-D for both large and small bleached regions for WT (blue squares) and *ras1*^*Q66L*^ (red circles). (F) Snapshots of FRAP simulations of wide (left) and narrow (right) regions.

We performed FRAP experiments of cells under different conditions to narrow down the possible transport mechanisms of Ras1 and regulators to the active patch. GFP-Ras1, which shows Ras1 localization independent of nucleotide binding, localizes along the whole cell cortex during the exploration phase [23]. Its intensity fluctuates around the cortex as its active form concentrates at the exploring patch, complicating FRAP analysis [23]. During interphase, GFP-Ras1 localizes all around the cell cortex but its active form is concentrated at the cell tips (Figs 1D and S1A) [23]. Thus, to monitor the dynamics of Ras1-GDP at the cortex, we bleached the sides of cells expressing GFP-Ras1 during vegetative growth, assuming that Ras1-GDP dynamics are similar during interphase and mating. The recovery of intensity was slower for wider bleached areas, consistent with diffusion-dominated recovery (Fig 2A). We fitted the FRAP data with a 3D model of membrane diffusion and cytoplasmic exchange that accounts for the geometrical features of the system, assuming that the concentration in the cytoplasm is uniform and constant (Fig 2A and 2B, Materials and Methods). A range of diffusion coefficients and exchange rates give curves that lie within the standard deviation of the experimental measurements (Fig 2E). To select from those values, we further fitted the recovery of the concentration profiles (Figs 2F and S1B and S1C), which favored the largest range of diffusion coefficients, with *D* = 0.15 μm^2^/s and exchange rates < 0.005 s^-1^.

To estimate the diffusion coefficient of Ras1-GTP, we performed FRAP of cells expressing GFP-Ras1^Q66L^ carrying a GTP-locked Ras1 allele, which decorates the whole cortex of interphase cells, Figs 2C and S1A. We repeated the same analysis procedure as for Ras1-GDP. We found the range of values of diffusion coefficient and exchange rates that provide fits for the total recovery in the bleached region versus time, within the experimental standard deviation (Fig 2D and 2E). To further narrow down the range of values in Fig 2E, we also fitted the individual recovery profiles (S1D and S1E Fig), which indicated a slower Ras1-GTP diffusion, *D* = 0.04 μm^2^/s and faster membrane dissociation rate, 0.02 s^-1^ as compared to Ras1-GDP. Cooperative interactions amongst activated Ras1 may further slow diffusion or dissociation in regions of high Ras1-GTP concentration, however these effects are harder to measure and, for simplicity, we neglect these effects in the model below.

**Fig 3:**
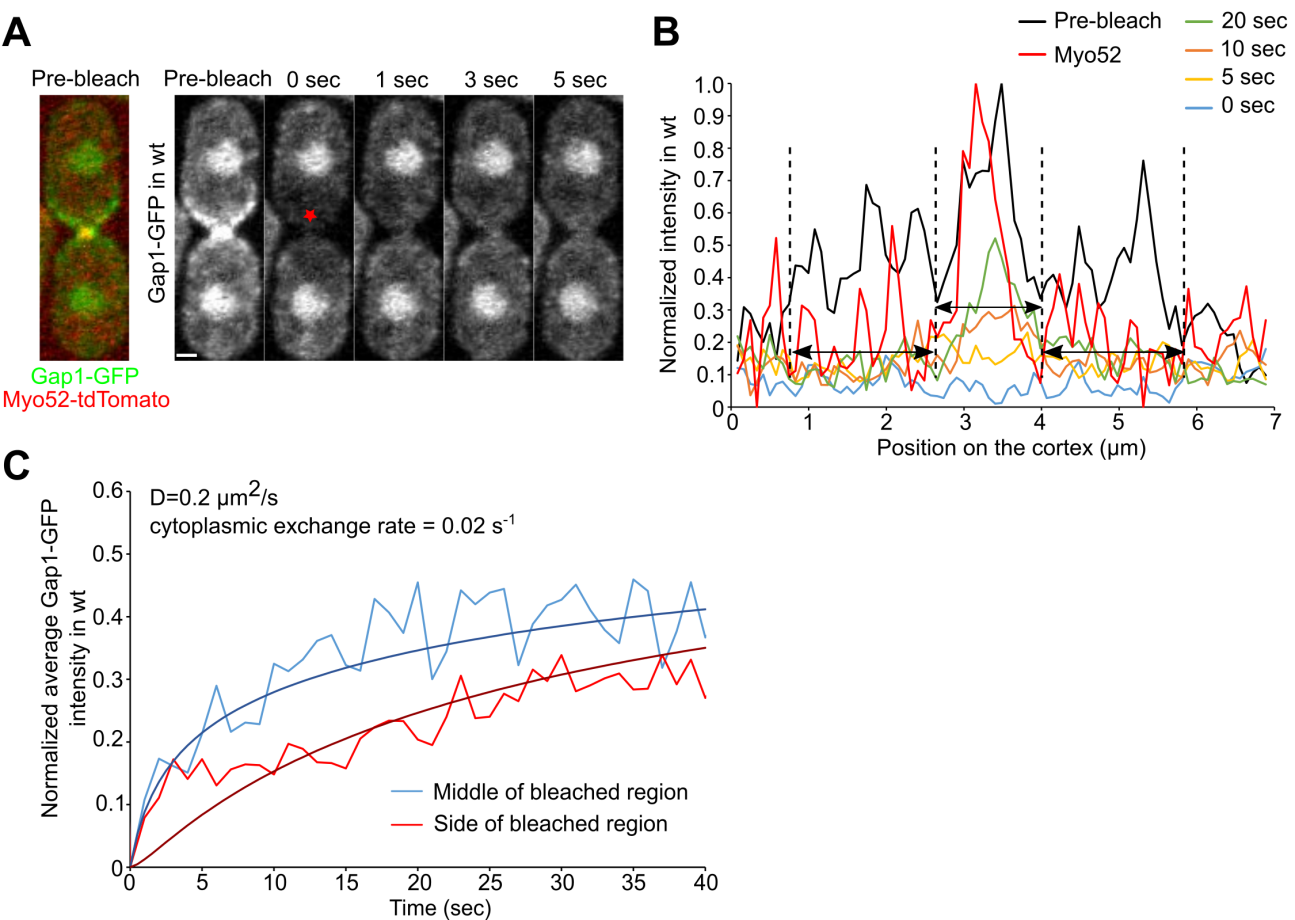
FRAP studies of Gap1 membrane diffusion. (A) Image of WT cells expressing Gap1-GFP and Myo52-tdTomato before bleaching and snapshots of FRAP of Gap1-GFP at the fusion focus (red star). The scale bar is 1 μm. (B) Intensity profile along the cell cortex at the indicated time points for the top cell in panel A. The Myo52-tdTomato intensity profile, as shown in panel A, is used to identify the center and the sides of the bleached region (black arrows). (C) Average recovery of Gap1-GFP for the cell shown in panel A at the middle and the sides of the bleached region as indicated by black arrows in panel B. The fits correspond to model with recruitment of Gap1-GFP to the cell tip (where the Myo52 zone is assumed to exist) with 50% recovery at long times. Good fits were obtained for $D=0.1-0.3{\mu m}^{2}s^{-1}$ and $r=0.015-0.025s^{-1}$.

Gap1 is recruited to the sites of Ras1 activation [23] and localizes to the zone of cell-cell contact during mating and to the tips of interphase cells, where Ras1 is active [23]. To measure Gap1 diffusion, we performed two types of FRAP experiments. In the first series, we bleached all of Gap1-GFP at the contact zone of pre-fusion paired cells expressing Gap1-GFP and Myo52-tdTomato (Fig 3A and 3B). In half of a total of 6 cells, the recovery of the middle of the fusion focus was faster than the side regions (the other half cells showed either small or no detectable difference) (Fig 3C). This behavior is consistent with a mechanism of recruitment of Gap1 at the patch center, followed by lateral diffusion to the cell sides. In the second series, we bleached half of the cell tip of vegetatively-growing cells expressing Gap1-GFP to monitor the diffusion process at the boundary between bleached and unbleached regions (S2A and S2B Fig). Using *D* = 0.2 μm^2^/s and dissociation rate 0.02 s^-1^ provides good fits for Gap1-GFP FRAP at the fusion site in mating cells (Figs 3C and S2E) as well as the smoothening out of the sharp intensity gradient at the boundary between the bleached and unbleached regions at the cell tip of the vegetatively-growing cells (S2C and S2D Fig). Thus the diffusion rate of Gap1 is similar to that of Ras1-GDP, to which it may remain bound after hydrolysis. These results support a Ras1-GTP focalization mechanism based on slow local activation coupled to a fast diffusing inhibitor (Gap1) and motivate a reaction-diffusion model we describe below.

### Development of a model that reproduces the exploratory Ras1 dynamics

**Fig 4:**
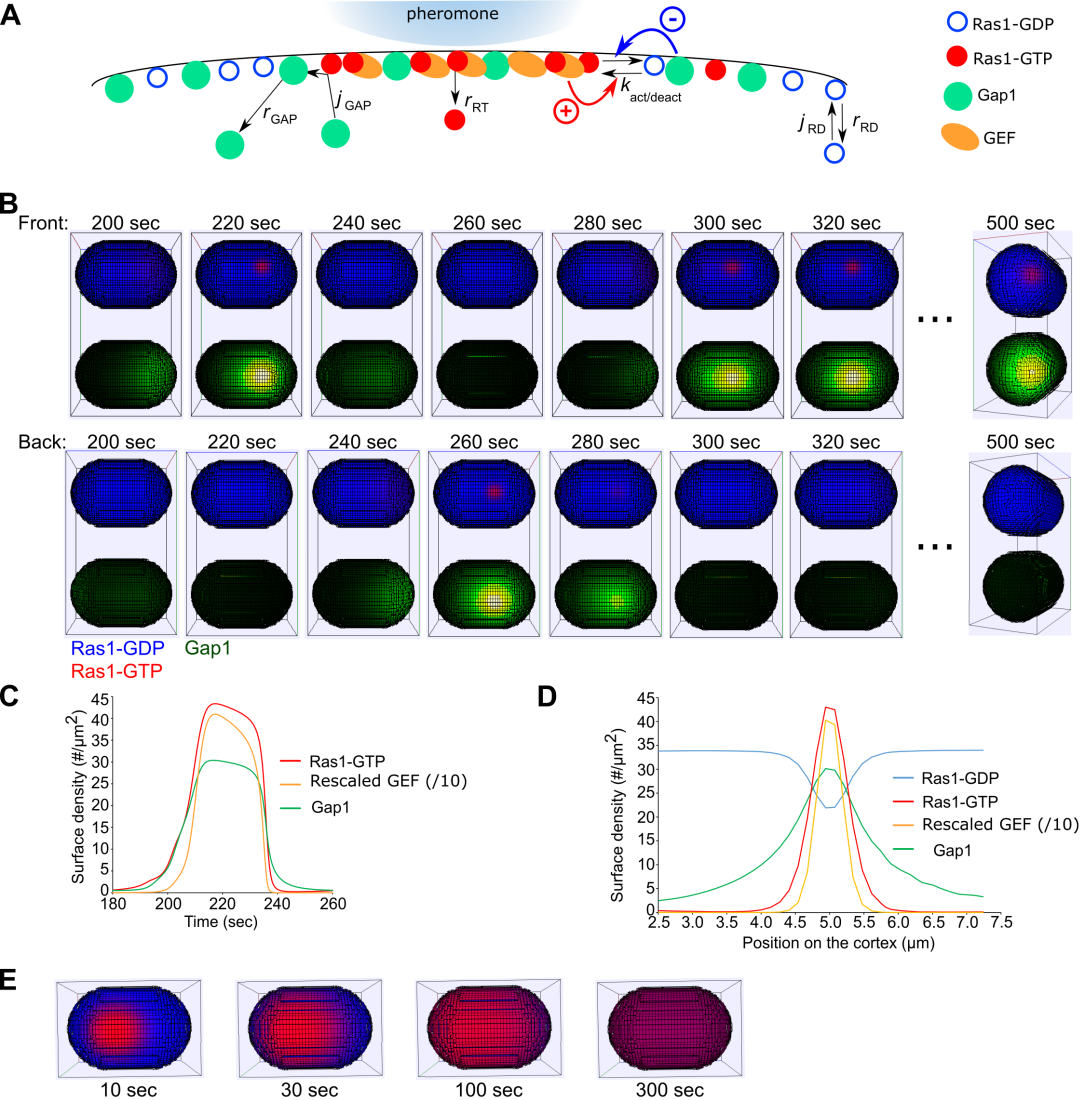
Model for Ras1 patch formation and exploration. (A) Schematic illustrating the model of Ras1 patch formation along the cell membrane. Stabilization can occur in a region with higher external pheromone concentration. (B) Snapshots of simulation showing appearance and disappearance of a patch on the surface of one cell. The top displays illustrate Ras1-GDP (blue) and Ras1-GTP (red). The bottom ones show Gap1 (green). Note that the first and second rows show views of the front and the upside down back of the same cell, illustrating the movement of a patch to a different cortical location. For this and following panels, the model parameters are from Table 1, unless otherwise indicated. (C) Simulated protein surface density at the center of a patch over time during patch appearance and disappearance. (D) Surface density profile over a 0.2 μm wide strip along the cell long axis going through the center of the patch at 220 sec for the simulation shown in panel B. (E) Elimination of negative feedback by setting the Gap1-dependent hydrolysis rate constant of Ras1-GTP equal to zero results in global activation of Ras1, similar to *gap1*Δ cells.

We developed a model based on a system of reaction-diffusion equations to study regulation of Ras1-GTP exploratory behavior and stabilization during fission yeast mating (Fig 4A). The model accounted for the diffusion and reaction of Ras1-GTP, Ras1-GDP and Gap1 along a 3D curved surface with surface densities $C_{RT},C_{RD}$, and $C_{GAP}$ that vary along the cell surface (see Material and Methods). These three components have diffusion coefficients $D_{RT},D_{RD}$ and $D_{GAP}$, and cytoplasmic exchange rates, $r_{RT},r_{RD}$ and $r_{GAP}$, which we have estimated from FRAP experiments as discussed previously. We assume that the cytoplasmic concentrations of Ras1-GDP and Gap1 are uniform in space and constant over time, but that there is a finite pool of GEF that provides an upper Ras1 activation limit [16]. Prior modeling work has shown how the combination of positive and negative feedbacks can lead to patch formation and patch oscillations of Cdc42-GTP [16, 21, 27]. We thus examined if similar mechanisms can underlie Ras1-GTP dynamics.

In the model, activation of Ras1 by GEFs is assumed to occur through an autocatalytic mechanism that has a functional form similar to the positive feedback in a winner-take-all mechanism of *S*. *cerevisiae* polarization [16]. This is motivated by the experimental observations of Ste6 (Ras1 GEF) colocalization with Ras1-GTP at the fusion focus [23] and the previously shown positive feedback between Ras1 and its own activator (Ste6) [25]. In this positive feedback mechanism, a finite amount of GEF in the system is assumed to be distributed with higher proportions at sites with higher active Ras1 concentration, i.e. we assumed that the density of Ras1 GEFs, $C_{GEF}$, is determined by $C_{RT}$.

A negative feedback is required to enable patch disappearance. We assume that Gap1 is recruited to the membrane through Ras1-GTP [28], where it hydrolyzes Ras1-GTP and diffuses laterally before dissociation. The Gap1 recruitment rate depends nonlinearly on Ras1-GTP concentration to generate oscillations, as in the GAP mechanism implemented to reproduce Cdc42 oscillations in *S*. *cerevisiae* [21]. This nonlinearity is supported by the experimental evidence that Gap1 full localization to sites of Ras1-GTP needs more than its GAP domain [23]. All membrane-bound Gap1 is assumed to be able to hydrolyze Ras1-GTP but we note that this is an approximation since one possibility is that Gap1 remains bound to Ras1-GDP, and thus inactive, after GTP hydrolysis.

By varying the unknown rate constants of activation and inactivation and using other parameter values from Table 1, we found that the model can reproduce exploratory behavior through the formation and disappearance of an active Ras1 zone (Fig 4B and S1 Movie). A zone initially forms when random fluctuations (implemented as stochastic noise) trigger the positive feedback to take off at a random location along the cortex. The Gap1 that is simultaneously recruited spreads by diffusion faster than Ras1-GTP, restricting the lateral expansion of the zone, contributing both to its finite size and to its eventual disassembly. Some Gap1 remains at the former zone site after Ras1-GTP decays (Figs 4C and S3), similar to experiment (Fig 6D in [23]). The spatial profile of the zone (Fig 4D) compares well with experiments, with a local accumulation of Ras1-GTP (as in Fig 3G in [23]) and a wider Gap1 compared to Ras1-GTP (as in Fig 6I in [23]). The exploratory period in the model is 60 sec, which is close to the observed 90 sec in wt mating mixture experiments [9].

**Table 1:** Model parameters.

Parameter	Value	Source
Cell length	6 μm	Measured the length of mating cells
Cell width	2 μm	Measured the width of mating cells
Cell volume	58.6 ${\mu m}^{3}$	
Number of pixels along long cell axis	45	
Number of pixels along direction perpendicular to long cell axis	20	
$D_{RD}$	0.15 ${\mu m}^{2}s^{-1}$	Estimated from FRAP experiments
$D_{RT}$	0.04 ${\mu m}^{2}s^{-1}$	Estimated from FRAP experiments
$D_{GAP}$	0.2 ${\mu m}^{2}s^{-1}$	Estimated from FRAP experiments
$r_{RD}$	0.001 $s^{-1}$	Estimated from FRAP experiments
$r_{RT}$	0.02 $s^{-1}$	Estimated from FRAP experiments
$r_{GAP}$	0.1 $s^{-1}$	Estimated from FRAP experiments
$k_{0}^{p}$	0.02 ${\mu m}^{2}s^{-1}$	Explored parameter space
$k_{1}^{p}$	5 ${\mu m}^{3}$	Explored parameter space
$k_{2}^{p}$	1000 ${\mu m}^{5}$	Explored parameter space
$k_{1}^{n}$	0.002 $s^{-1}$	From in vitro hydrolysis experiments [23]
$k_{2}^{n}$	0.1 ${\mu m}^{2}s^{-1}$	Larger than in vitro measurements [23], to generate Ras1 patch disassembly rates comparable to experiments
$k_{3}^{n}$	100 ${\mu m}^{-2}s^{-1}$	Chosen such that the maximum Gap1 density is of order the Ras1-GTP density
$C_{sat}$	60 ${\mu m}^{-2}$	Chosen such that negative feedback saturates near the maximum of Ras1-GTP density
*h*	2	The lowest cooperativity for negative feedback
${E_{c}}^{tot}$	100 molecules	Ste6 could not be functionally tagged [23]. We assumed the number is similar to that of Efc25 during vegetative growth [29].
$C_{RD}$ (far from patch)	38 ${\mu m}^{-2}$	Based on estimate of 7000 Ras1 molecules per cell, half of which on the cell surface.
$j_{RD}^{p}$	0.038 ${\mu m}^{-2}s^{-1}$	Adjusted to give $C_{RD}$ *=* 38 ${\mu m}^{-2}$ using $j_{RD}^{p}={r_{RD}C}_{RD.}$ This equation applies far from the patch where Ras1-GDP recruitment to cell membrane balances dissociation into cytoplasm.

Our model provides an explanation for the uniform cortical activation and absence of active Ras1 patch in *gap1*Δ cells [23]. Upon removal of the Gap1-dependent hydrolysis of Ras1-GTP from the model (while keeping other parameters fixed as in Table 1), a zone is formed that keeps spreading until covering the whole cortex (Fig 4E). Thus, a minimum threshold of Gap1-dependent hydrolysis of Ras1-GTP is required to stop the spread of the patch into a homogenous state.

**Fig 5:**
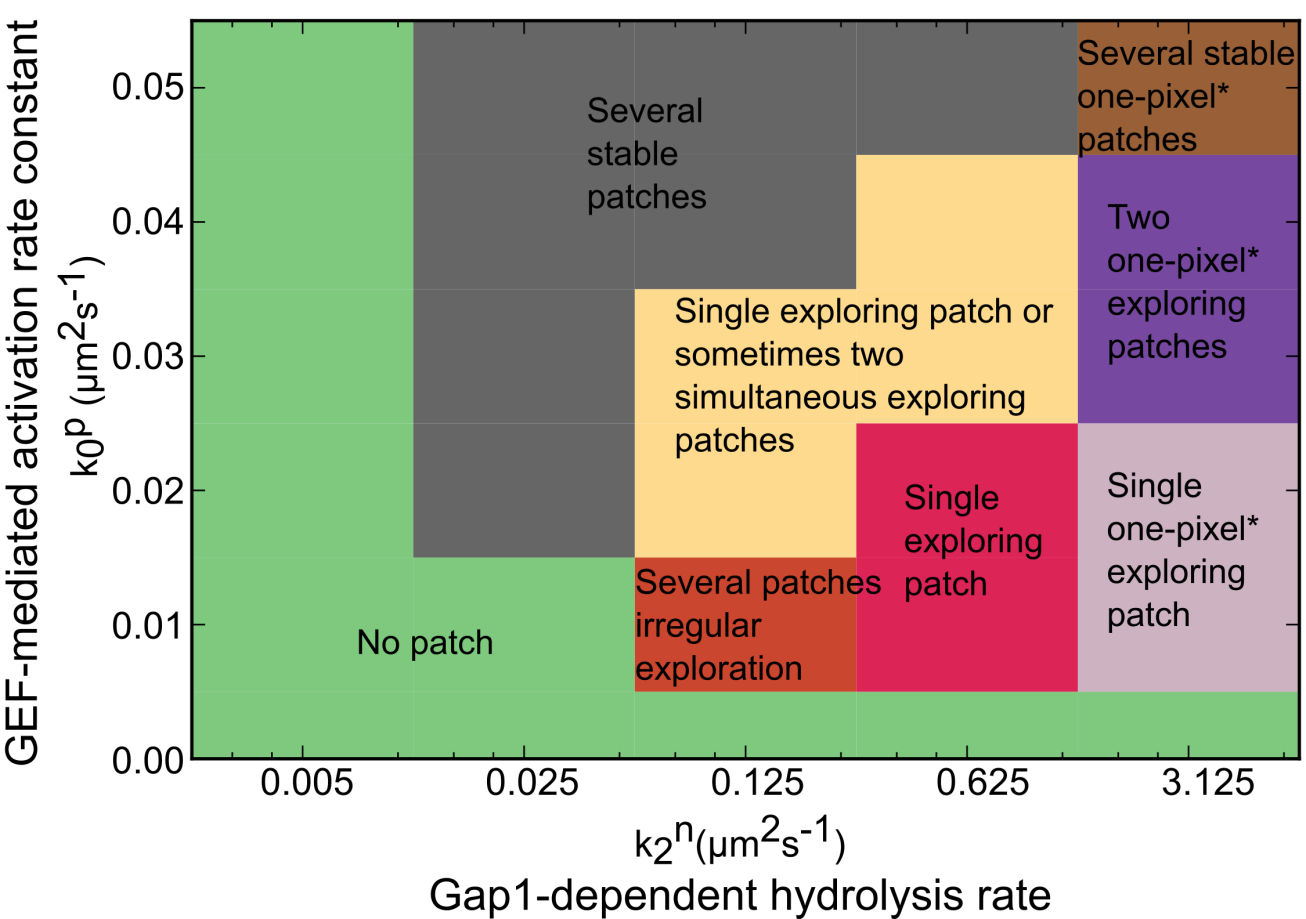
Regions of dynamical behavior. Behavior observed in simulations for different values of $k_{0}^{p}$, the GEF-mediated activation rate constant of Ras1-GTP, and $k_{2}^{n}$, the Gap1-dependent hydrolysis rate constant of Ras1-GTP. Other free parameters were kept as shown in Table 1. The yellow region represents simulations that mostly show a single patch oscillating however sometimes there are two patches that form simultaneously and then disappear at the same time or one after the other. One pixel* is one voronoi cell of the simulated surface.

The behavior of the system in parameter space, varying the main coefficients of the positive feedback (the GEF-mediated activation rate constant of Ras1-GTP), and negative feedback (the Gap1-dependent hydrolysis rate constant of Ras1-GTP) is shown in Fig 5. The figure shows the region with single patch exploratory behavior surrounded by a region that lacks patches and other regions that may contain multiple stable or fluctuating patches. The region that lacks patches shows two different qualitative behaviors: first, when the positive feedback parameter is too small to initiate patch formation; second, when the negative feedback is too small to keep the patch from spreading all over the surface towards a homogeneously activated state. The behavior of the system as a function of saturation coefficient in recruitment of Gap1 and Gap1-dependent hydrolysis rate of Ras1-GTP is plotted in S4 Fig, which shows a similar structure with a region of single patch exploratory behavior surrounded by regions of different dynamics.

### Transition from exploration to patch stabilization

We studied the mechanisms by which our model can reproduce the transition from exploration to a stable zone, as occurs in the cell’s response to sensing of external pheromone. This change in the dynamical behavior of the system upon pheromone sensing could occur by three possible mechanisms: (1) a uniform change of the Ras1 rates constants of our model across the cell cortex, (2) a local change involving differential regulation of the Ras1 rate constants around the Ras1 patch site, or (3) a local change in the Ras1 rate constants around the region of high pheromone concentration. The last two mechanisms may be equivalent because pheromone receptors are locally active at the patch location, though prior data cannot distinguish whether, as in the second option, the patch brings receptors along to probe for local signal, or whether, as in the third option, receptors may be present and activated elsewhere at the cortex and promote patch stabilization if it forms at this location [11]. We note that this regulation likely involves additional factors not included explicitly in our model such as activated receptors, actin or MAPK signaling components [10, 30, 31].

**Fig 6:**
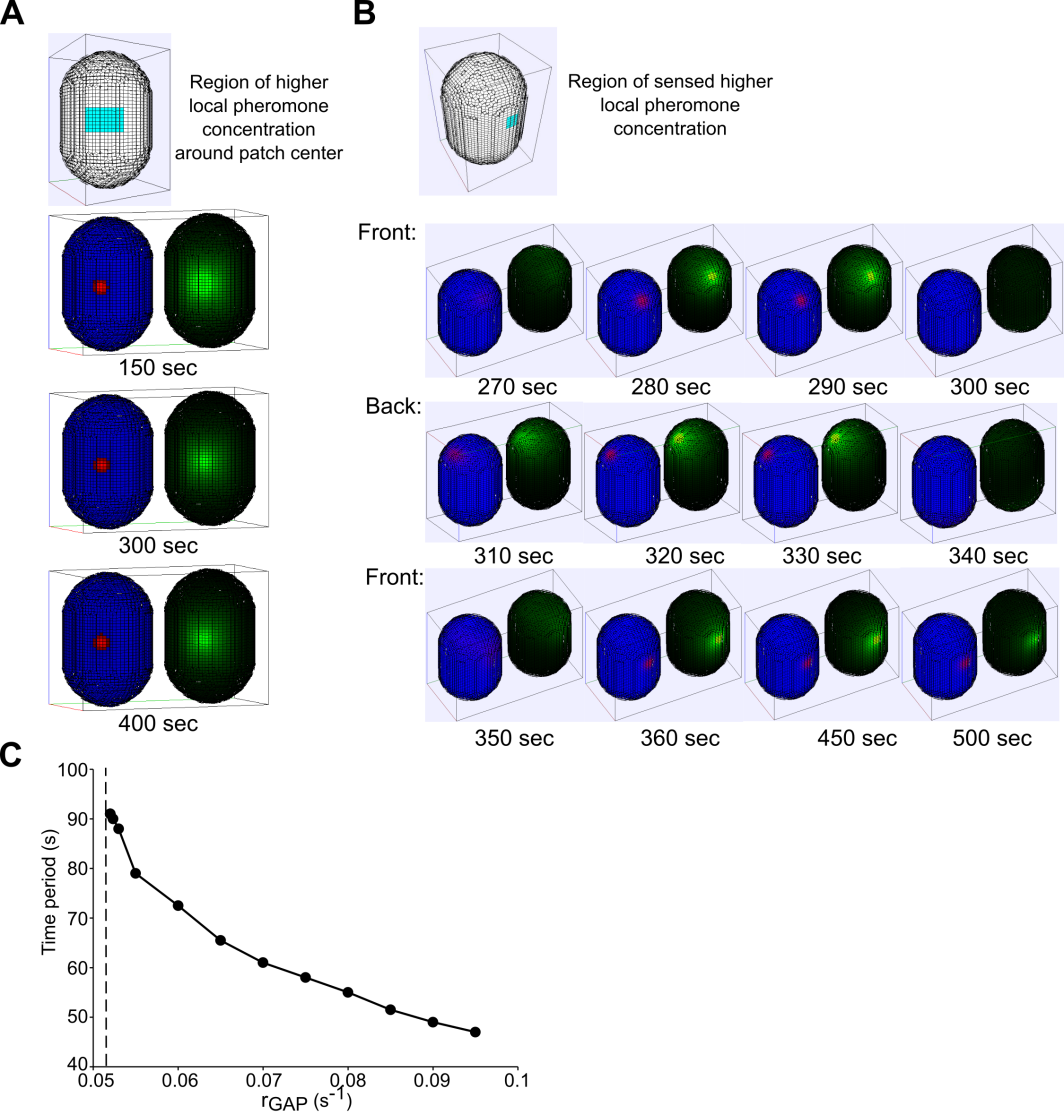
Patch stabilization through changes in model parameters. (A) Simulation of local increase in positive feedback over a region around the patch center (Ras1 activation rate constant $k_{0}^{p}$increased by 1.25 times over cyan colored region without any change compared to Table 1 values in white region) results in stabilization of the exploring patch. (B) Simulation of local increase in positive feedback at a specified fixed region on the membrane (Ras1 activation rate constant $k_{0}^{p}$increased by 1.2 times over cyan colored region without any change compared to Table 1 values in white region). Snapshots show patch exploration and stabilization at the site of local positive feedback increase. Note that middle row shows back-side upside-down snapshots. (C) Time period of exploring patch appearance and disappearance increases with decreasing detachment rate constant of Gap1, $r_{GAP}$. It converges to 91 seconds before further decrease of $r_{GAP}$ below dashed line results in formation of a stable patch.

The three mechanisms of patch stabilization described in the preceding paragraph were studied in simulations. A uniform change in the positive feedback rate constant $k_{0}^{p}$ or negative feedback $k_{2}^{n}$ across the cell cortex (around the reference values in Table 1) does not readily produce single stable patches but rather states with either none or multiple patches (Fig 5). However, a local increase of the positive feedback rate constant over a region on the cell membrane around the center of the exploring patch results in stabilization of the patch at that location (Fig 6A). This could result through locally stimulated receptors that regulate Ras1 positive feedbacks to stabilize the patch when it happens to form at a region of higher pheromone concentration [11]. A relatively small increase in the rate constants of the positive feedback over a fixed region on the cell membrane (corresponding to higher external pheromone) can also result in patch stabilization on that site after a few rounds of exploration (Fig 6B). A larger local increase causes the zone to form directly at that location. In conclusion, the model allows patch stabilization through mechanisms 2 and 3.

Another way to obtain a single stable patch is to decrease the detachment rate of Gap1 $r_{GAP}$ (Fig 6C) or decrease the saturation coefficient of the Gap1 recruitment rate $C_{sat}$ and Gap1-dependent hydrolysis rate constant $k_{2}^{n}$ (S4 Fig). Whether these parameter changes occur locally around the patch or uniform across the surface is not as important because Gap1 is recruited at the patch following Ras1-GTP.

In prior experiments with external P factor sensed by cells lacking the P factor protease, the period of a single exploratory zone increased with pheromone concentration, which led to a model of partner selection through mutual stimulation [11]. We found that the patch lifetime in our model did not change significantly during the transition from exploration to stabilization through local positive feedback regulation (around the reference values of Table 1): as the positive feedback rate constants were increased locally, or decreased elsewhere on the cortex, the patch abruptly transitioned from exploratory dynamics with lifetime ~ 60 s to a single stable non-exploring patch. Similar abrupt transition (exploration with 60 s lifetime to stable patch) can be observed with a local decrease in the Gap1-dependent rate hydrolysis ($k_{2}^{n}$).

Interestingly, we found that decreasing $r_{GAP}$ globally lead to an increase of the lifetime of the exploring patch (Fig 6C). If such a mechanism underlies the control of Ras1 patch lifetime, then we would expect that the longer the patch lifetime, the higher the Gap1 concentration accumulated locally. The observed increase in Gap1 intensity at the fusion focus compared to an exploring patch [23] is consistent with the Gap1 membrane residence time playing a role in regulation of the patch lifetime in response to sensed pheromone concentration. We note that this is not the only possible mechanism leading to an increase in Gap1 intensity.

In conclusion, the model suggests that regulation of both positive and negative feedbacks is required for the observed dynamical response to pheromone in experiments (i.e. exploration to stabilization). We note that we cannot exclude the possibility that additional adaptation mechanisms [32] (in which the patch duration under high pheromone is related to the time required for the system to adapt back to the exploratory state) or other regulatory mechanisms, such as multi-step positive or negative feedbacks that couple to the sensing and Cdc42 system, may also regulate the patch period during the transition to a single stable zone.

### Regulation of patch size

The model further suggests how positive and negative feedbacks regulate the width of the Ras1-GTP zone, which may be part of the partner distance sensing mechanism. Indeed, it has been recently demonstrated that Ras1-GTP patch at the fusion focus is narrower in comparison to the exploratory patch (Fig 3G in [23]). In this section we study in more detail how feedback mechanisms regulate patch size in our model, and test them experimentally.

**Fig 7:**
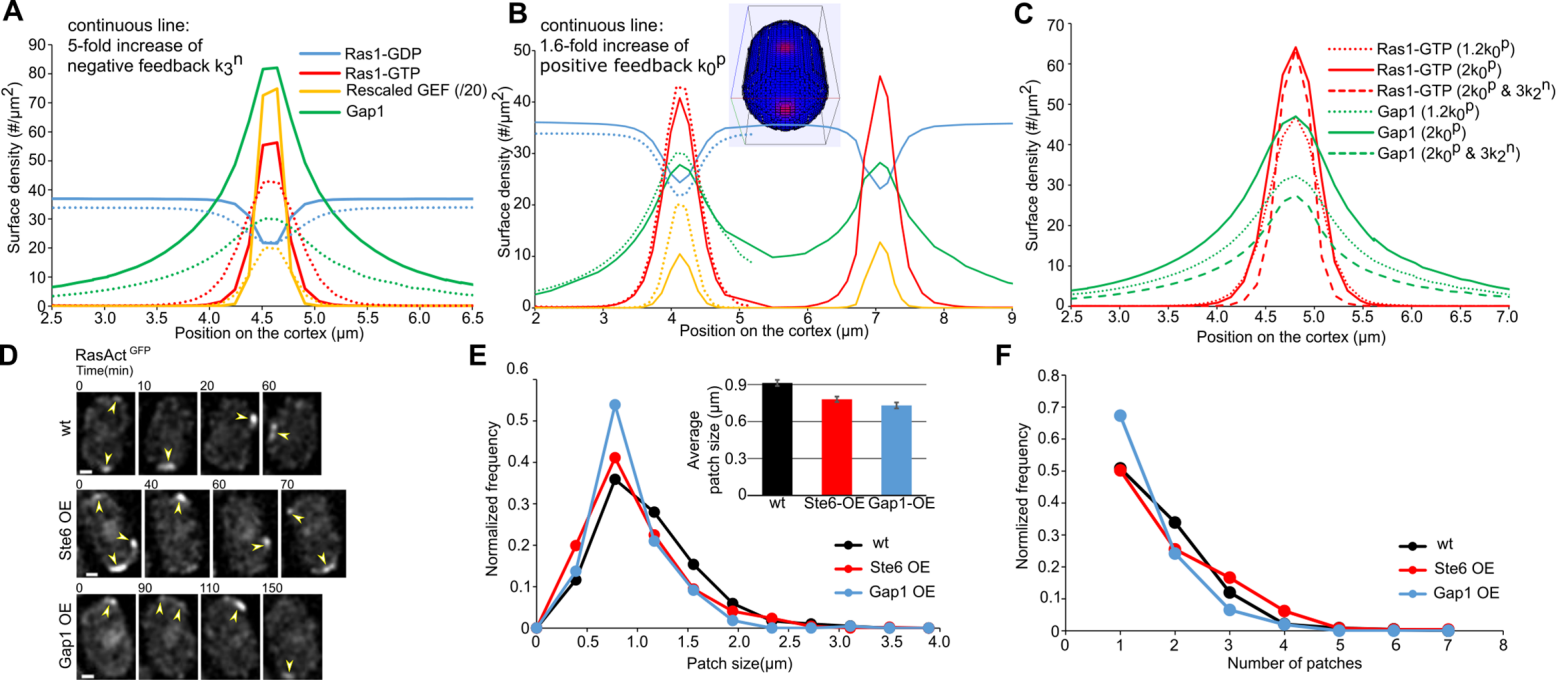
Regulation of a stable patch size through positive and negative feedbacks. (A) Surface density profile over a 0.2 μm wide strip along the cell long axis going through the center of an exploring patch at its peak, for overall stronger negative feedback. Solid lines: Increase of Gap1 recruitment rate constant $k_{3}^{n}$by 5 times; Dotted lines: Reference curves from Fig 4D (values from Table 1). (B) Surface density profile over a 0.2 μm wide strip along the cell long axis going through the center of two aligned patches peaking simultaneously, for stronger positive feedback. Solid lines: Increase of Ras1 activation rate constant $k_{0}^{p}$by 1.6 times. Dotted lines: reference curves from Fig 4D. (C) Surface density profile over a 0.2 μm wide strip along the cell long axis going through the center of a patch stabilized via stronger local positive feedback. Curves show effect of change with respect to values of Table 1: (i) increase of Ras1 activation rate constant $k_{0}^{p}$by 1.2 times; (ii) Increase of $k_{0}^{p}$by 2 times; (iii) and increase of $k_{0}^{p}$by 2 times and Gap1-dependent hydrolysis rate constant $k_{2}^{n}$by 3 times, the latter over a 1.5 fold larger area than the cyan color region shown in Fig 6B. (D) Snapshots of RasAct^GFP^ exploratory patch during early stages of mating in WT, Ste6 overexpression and Gap1 overexpression cells also expressing Myo52-tdTomato. The scale bar is 1 μm. Shown examples are not necessarily consecutively observed patches. (E) Patch size in WT (*n* = 404 patches in 23 cells), Ste6 overexpression (*n* = 467 patches in 28 cells) and Gap1 overexpression cells (*n* = 219 patches in 24 cells). Inset shows the average patch size and standard error calculated for WT (0.91±0.02μm), Ste6 overexpression (0.78±0.02μm) and Gap1 overexpression cells (0.73±0.02μm). The two sample t-test between WT and the mutants shows mean patch sizes are different at 0.01 significance level. (F) Normalized frequency of observed simultaneous patches in WT, Ste6 overexpression and Gap1 overexpression cells. We note that the patch intensity decreases in Gap1 overexpression cells (S6A Fig) while our threshold for patch detection is the same as wt cells; thus we cannot exclude the possibility of more patches in Gap1 overexpression cells below the detection threshold.

Increasing the Gap1 recruitment rate (parameter $k_{3}^{n}$) or the Gap1-dependent hydrolysis rate of Ras1-GTP (parameter $k_{2}^{n}$), with respect to the reference values of the Table 1, produces smaller patches (Figs 7A and S5A). The patch size gets smaller until reaching the minimum size limit of the simulations, which is equal to one polygonal cell on the surface mesh (one voronoi cell) (Fig 5). As discussed above, local recruitment of Gap1 that spreads in the vicinity of Ras1-GTP by diffusing faster than Ras1-GTP, restricts patch size. As a consequence, larger net hydrolysis rates lead to narrower patches, containing a higher concentration of Ras1-GTP, with approximately the same amount of GEF distributed over a narrower area.

An increase in the total GEF amount (parameter $E_{c}^{tot}$) or a uniform increase in the positive feedback rate constants ($k_{0}^{p}$ or $k_{1}^{p}$ and $k_{2}^{p}$) along the cell membrane results in the formation of two patches that compete within the appearance and disappearance time period (Figs 7B and S5B and S5D and S2 Movie). This is due to the fact that the stronger positive feedback rates, the result of a change in the above parameters, lead to higher nucleation rate during the process of a prior patch inactivation; this increases the likelihood that two patches competing for a limited pool of GEF start growing within a short time of one another, such that none of them has enough time to become dominant [33]. The system transitions to a very slow-competing phase with two or more stable zones upon further increase of the above parameters ($E_{c}^{tot},k_{0}^{p},k_{1}^{p}$ and $k_{2}^{p}$) (Figs 5 and S4).

We also explored the dependence of a stable patch size to changes in the positive and negative feedback rate constants (Fig 7C where $k_{0}^{p}$ was locally increased by 20% compared to the Table 1 to get a stable patch). Such a stable patch could be the result of stabilization after local pheromone sensing during the process of exploration or a patch at the tip of a shmoo growing closer towards that of a partner cell. We found that further increase in $k_{0}^{p}$ lead to higher concentration of Ras1-GTP and Gap1 and marginally narrower patch. Additional increase of the Gap1-dependent hydrolysis rate ($k_{2}^{n}$) caused the patch to become narrower, as in Fig 7A, however in this case the concentration of Ras1-GTP did not increase significantly.

In summary, we find that increase in negative feedback rates makes the patch narrower with the intensity of Ras1-GTP staying the same or increasing depending on the reference state (i.e. exploring or stable patch). Increase in the positive feedback can cause formation of multiple patches or cause single patches to recruit more Ras1-GTP and Gap1, becoming marginally narrower.

To test our prediction about patch size regulation through positive and negative feedback we measured the size of exploratory Ras1-GTP patches in cells expressing RasAct^GFP^ and Myo52-tdTomato in wt and mutants overexpressing Gap1 or the GEF Ste6 (Fig 7D). By imaging every 10 min through a medial focal plane, we identified exploring cells in which a RasAct patch appeared in different position along the cortex. We identified patches as regions of the cell cortex with intensity above the cytoplasmic background (three times the standard deviation of the cytoplasmic region excluding the nucleus; the cytoplasmic intensity and its standard deviation was similar in all three cases, see Materials and Methods and S6 Fig). We did not use Myo52 localization as a criterion for patch detection since RasAct patches did not always colocalize with Myo52 (though they were frequently found together). Since RasAct has a spottier distribution compared to other patch markers such as Scd2, RasAct dots that were close to one another (of order 2 pixels = 260 nm) were counted as a single patch (Fig 7D). Cells occasionally contained more than 1 patch, each one of which could be at different stages of appearance and disappearance. We quantified the distributions of size and number of exploratory patches in Fig 7E and 7F (averaged over fluctuations in z position with respect to the focal plane; See Materials and Methods).

The experimental results in Fig 7 are in agreement with the trends predicted by our model. The average exploratory patch size in Gap1 overexpressing cells, 0.73±0.02μm (SEM) was smaller than 0.91±0.02μm in wt cells (Fig 7E, inset) and the number of patches per cell was less compared to wt cells (Fig 7F). This trend is in agreement with Fig 7A (increase in negative feedback parameter $k_{3}^{n}$), as well as Fig 5, which shows that increase in the negative feedback parameter $k_{2}^{n}$ moves a system originally in the single patch oscillation region towards a region of narrower patches and away from a region with multiple patches. The patch size in Ste6 overexpressing cells 0.78±0.02μm (SEM) decreased compared to wt cells, in agreement with Fig 7C (increase in positive feedback rate constant $k_{0}^{p}$ or total GEF amount $E_{c}^{tot}$ (S5C Fig)). The model results in Figs 7B and 5 predict an increase in number of patches with an increase in the total amount of GEF or uniform increase of positive feedback rate constant $k_{0}^{p}$. A larger number of patches on average was indeed observed in Ste6 overexpressing cells (Fig 7F) though the relative magnitude of the change is small.

Though the experiments agree with model predictions, we note that patch size may be controlled by multiple factors. First, we should be cautious about interpreting the results of Ste6 overexpression in terms of changes in model parameters because the mechanism for Ste6 expression, activation and interaction with the pheromone signaling pathway is not fully resolved [7]. Second, we note that we did not consider the interaction between Cdc42 and Ras1 [34] that might form feedbacks that regulate the patch size. Finally, the actin cytoskeleton, endocytosis and exocytosis also likely contribute to the regulation of patch size especially in the final stages of fusion when the pheromone-induced formin Fus1 assembles the fusion focus [35]. We note that at these late stages, the curvature of the cell tip may also play a role. Bonnazi et al. [36] showed that the curvature-dependence of Cdc42 zone size required formin nucleated cortical actin cables and fusion of secretory vesicles transported along the actin cables. In our model, curvature had no drastic effect: variation of the tip radius of curvature from 1.8 to 2.3 μm while keeping the total cell surface area fixed gave Ras1-GTP and Gap1 patch sizes that varied by 1–4 Voronoi cells (for stable patches generated using $C_{sat}$ = 20 μm^-2^ and $k_{2}^{n}$ = 0.01 μm^2^s^-1^, see S4 Fig., and other parameters as in Table 1; this small effect depended on the different Voronoi cell discretization at different curvatures).

## Discussion

In this study we showed that Ras1-GTP patch dynamics during mating in fission yeast cells can be modeled as reaction-diffusion system on the cell membrane with combined positive and negative feedbacks. We used FRAP experiments to estimate the diffusion coefficients of the main components in the system. The measured value of Ras1-GDP diffusion coefficient is similar to that of Cdc42-GDP membrane diffusion in fission yeast [37]. The slower Ras1-GTP diffusion is consistent with the slower diffusion coefficient of Cdc42-GTP compared to Cdc42-GDP [37]. Our estimation for Ras1-GDP membrane diffusion is twice the measured value for GFP-Ras2 membrane diffusion in budding yeast [38]. A very slow cytoplasmic exchange rate, comparable to our estimate, was measured for both single and double lipidated GFP-Ras2 [38]. Our values are also comparable to measurements of lateral diffusion of H-Ras and K-Ras proteins in mammalian cells [39, 40]. The measured slower Ras1-GTP diffusion is also in agreement with the observed reduction in membrane diffusion of active H-Ras and K-Ras in rat cells in comparison to inactive forms [41]. In mammalian cells, Ras proteins organize into nanodomains with distinct dynamics [42, 43]; here our measurements represent an average of diffusion through such possible nanodomains.

Using the measured values of diffusion coefficients, the model reproduces an exploratory patch with a lifetime comparable to experiments, and allows a switch from exploration to a stable patch by changes in the model’s rate constants. A patch forms in our model because Gap1 is recruited through locally self-amplifying Ras1-GTP. Due to its higher diffusion coefficient, it spreads further than the Ras1-GTP patch and limits the spread of the Ras1-GTP patch. Due to limited amount of GEF in the cell, autocatalytic activation of Ras1 slows down and saturates as the cytoplasmic pool of GEF gets depleted. The patch then starts to decay when the locally accumulated Gap1 inhibitor inactivates Ras1-GTP, releasing some of the GEF. This process allows a competing new patch to start elsewhere in the cortex, drawing the GEF away from the old patch. A local increase in the positive feedback rate constants above a certain threshold, which could occur by local pheromone sensing, stabilizes the patch against competition from other patches that have smaller positive feedback rate constants.

In the model, a balance of positive and negative feedback reactions, diffusive flux along the cortex and between cortex and cytoplasm is established, which regulates the patch size. We find that the patch size depends on the magnitude of Ras1-GTP inhibition. Increase in Gap1 recruitment rate to the cortex results in smaller patches. This prediction was confirmed by the experiments with overexpression of Gap1.

We also described the dynamical behavior of the model surrounding the state of single patch exploration (Figs 5 and S4), which may be observable with mutant alleles. Here we kept the values of diffusion coefficients equal to those we extracted from experimental data. We note another possible dynamical behavior: when the diffusion coefficient of Gap1 becomes sufficiently smaller than the reference value, the activation process is not confined to a patch and the system can transition to travelling wave behavior.

The mechanism of gradient sensing in mating fission yeast is an example of a cell system exhibiting a state of active exploratory fluctuations or oscillations that stabilize along a direction upon sensing of external signal [1, 44–46]. Since the exploratory state in this system consists of a single polarity patch, this process can also be described as a system that establishes polarity prior to gradient direction sensing [9, 10]. At the single cell level, exploration here works as part of the search mechanism for an optimal configuration (similar to finding a potential well in a wide and flat energy landscape), while Cdc42 fluctuations and oscillations during the transition from monopolar to bipolar growth in fission yeast have been described as facilitating the escape from an asymmetric state towards a symmetric polarization [27] (similar to crossing a barrier separating two potential wells). At the level of a cell population, exploratory partner search has been proposed to aid the population reach an optimal number of mating pairs [11] (in this case, over a landscape with many barriers and local minima).

Gradient sensing mechanisms in other cell systems have also been modeled with reaction-diffusion equations and related methods. In mating conditions, budding yeast cells form a Cdc42-GTP patch that wanders around the cell cortex, executing a biased random walk rather than appearance and disappearance. The patch moves upstream of the pheromone gradient and stabilizes close to opposite mating type [12]. Two recent studies [10, 13] proposed mechanisms for this type of patch motility, which differ to what we considered in our work. McLure et al. provided a mechanism based on local exocytosis contributing to patch displacement. In the mechanism of Hegemman et al. patch lateral motility depends on stochastic fluctuations: patch formation depends on autocatalytic activation of Cdc42 (based on a prior model [47]); a secondary positive feedback between actin-based receptor trafficking and Cdc42 [10, 47] results in biased motion up the pheromone gradient and stabilization once a high level of Cdc42-GTP is achieved. By contrast, patch formation and disappearance in our model relied on Turing-type mechanisms previously proposed for interphase budding yeast polarization and oscillations: from these studies we borrowed the mechanisms of competition of finite GEF [16] and Gap1 inhibition [21] used to model transient polarity patch oscillations.

Gradient sensing of chemoattractant has been widely studied in motile cells, such as amoebas, neutrophils, neurons and fibroblasts [1, 10, 45]. In many cases, the process of response to the gradient has similarities to the exploration and stabilization process of fission yeast mating patch. For example, upon starvation conditions, *D*.*discoideum* cells start the process of multi-cell aggregate formation by first breaking symmetry by forming filopodia and pseudopodia along many directions; those that happen to form in the direction of a diffusible external cue (cAMP secreted by other cells) win over the rest [48]. Thus, models developed for these systems share common features to our study.

Meinhardt (1999) proposed a model with a generic autocatalytic fast diffusive activator and a slow diffusive long-range inhibitor model, which is able to generate a stable Turing pattern. Addition of an extra generic inhibitor, which acts locally on the activator maximum peak to deactivate it leads to the generation and subsequent decay of a local activator maxima [15]. These local maxima are more likely to form along the direction in which the positive feedback rate constants have higher values (analogous to the process shown in Fig 6B for our model). A problem with this model is that it does not exhibit perfect adaptation, which inspired the development of Local Excitation, Global inhibition model [1]. The LEGI model has a fast acting local activator and a slow global inhibitor responding in direct proportion to an external signal. This model achieved perfect adaptation to signal gradient as well as high degree of sensitivity to changes in the signal gradient. However this model in turn needs additional components to generate significant amplification of an external stimuli and cannot account for persistence of polarity after the external signal is removed, as occurs in neutrophils and fibroblasts [45, 48]. More recent models have combined the LEGI model with an actin excitable system [32], to account for the process of adaptation as well as the existence of multiple layers of the process of cell polarization (accumulation of signaling components, actin polymerization, etc). Such models can further explain how *D*.*discoideum* cells in which actin polymerization was inhibited, were still capable of accumulating signaling proteins without pseudopodia or filopodia in response to the external cue gradient, as well as adapting to uniform increase of external signal [1].

Ras has been proposed to be involved in the signaling network providing perfect adaptation in *D*.*discoideum* (consistent with the LEGI model), with positive and negative feedback forming a parallel incoherent feedforward loop in response to external signal [49]. This is a different Ras signaling network connectivity compared to our model: in our case negative feedback follows a positive feedback that self-amplifies to form an exploratory patch, even without an increase of external input. While little is known about the adaptation properties of mating fission yeast, the fact that increase of external pheromone leads to measurable changes in patch lifetime [11] suggests that adaptation is either not perfect or else it occurs over times much longer than the patch lifetime. Despite this difference, we nevertheless anticipate a multilayer mechanism (analogous to *D*.*discoideum*) to exist in fission yeast. This requires further modeling work in future studies that include the contributions of Cdc42 and the actin cytoskeleton in addition to Ras1.

## Materials and methods

### Strains, media, and growth conditions

Strains used in this study are listed in Table 2. Standard genetic manipulation methods for transformation and tetrad dissection of *S*. *pombe* were used. For FRAP (Fluorescence Recovery After Photobleaching) experiments during exponential growth, cells were grown in Edinburgh minimal medium (EMM) supplemented with amino acids as required. For FRAP experiments and microscopy of cells during the mating process, liquid or agar minimal sporulation medium without nitrogen (MSL-N) was used [50, 51].

**Table 2:** Strains used in this study.

YSM3045	*h- GFP-ras1*	[23]
YSM3082	*h+ gap1-GFP-kanMX*	[23]
YSM3087	*h90 gap1-GFP-kanMX myo52-tdTomato-natMX*	[23]
YSM3119	*h- GFP-ras1- Q66L*	This study
YSM3120	*h90 leu1-RasAct-3GFP-leu1+ ura4-kanMX-Pnmt1-ste6-ura4+*	This study
YSM3121	*h90 leu1-RasAct-3GFP-leu1+ ura4-kanMX-Pnmt1-gap1-ura4+*	This study
YSM3122	*h90 leu1-RasAct-3GFP-leu1+*	This study

Gene tagging was performed at endogenous genomic locus at the 3’ end, yielding C-terminally tagged proteins, as described [52]. N-terminal tagging of Ras1 with GFP was performed as in [11]. Gene tagging was confirmed by diagnostic PCR for both sides of the gene.

Construction of fission yeast strains expressing overexpression of Ste6 and Gap1 (*Pnmt1-ste6* and *Pnmt1-gap1*) was done by integration of *ste6* and *gap1* under *nmt1* promoter at the *ura4+* locus. First, *kanMX-Pnmt1* fragment was excised from plasmid pSM647 (*pFA6a-kanMX6-Pnmt1*) through digestion with XmaI and EcoRI and ligated into similarly treated pAV133 (pJK211, a kind gift from Dr. Aleksandar Vjestica, UNIL) to generate plasmid pSM2106 (*pJK211-kanMX-Pnmt1*); second, *ste6* was amplified from genomic DNA with primers osm4924 (5’- tccccccgggATGAGGTTTCAAACGACCGCAATAAG) and osm4925 (5’- tccccgcggTCAAAAAATGCCAGAATCAATTAGC), digested with XmaI and SacII and ligated to similarly treated pSM2106 to generate plasmid pSM2111 (*pJK211-kanMX-Pnmt1-ste6*); *gap1* was amplified from genomic DNA with primers osm4918 (5’- tccCCCGGGATGACTAAGCGGCACTCTGGTACC) and osm4919 (5’- aaggaaaaaagcggccgcgTTACTTTCGTAAAAACAATTGTTC), digested with XmaI and NotI and ligated to similarly treated pSM2106 to generate plasmid pSM2110 (*pJK211-kanMX-Pnmt1-gap1*). Finally, pSM2110 and pSM2111 digested with AfeI were stably integrated as a single copy at the *ura4+* locus in the yeast genome. In primer sequences, restriction sites are underlined.

Construction of strains to visualize the constitutively active *ras1*^*Q66L*^ allele was done by integration of *GFP*-*ras1*^*Q66L*^ at the endogenous *ras1* locus. First, pSM1221 (*pREP41-Pras1-GFP-ras1*, [11]) was subjected to site directed mutagenesis with primers osm2167 (5’- GTATTGGACACGGCCGGT**CTA**GAGGAATATTCCGCTATG) and osm2168 (5’- CATAGCGGAATATTCCTC**TAG**ACCGGCCGTGTCCAATAC) to generate plasmid pSM1392 (*pREP41-Pras1-GFP-ras1*^*Q66L*^). Second, pSM1392 digested with PstI and XmaI was stably integrated as single copy at the *ras1* locus in the yeast genome, through transformation of a *ras1*::*ura4+* strain and selection on agar plates containing 5-Fluoroorotic Acid (5-FOA). In primer sequences, inserted mutations are bold.

### Microscopy and FRAP assays

The DeltaVision platform (Applied Precision) described previously [53] was used for time-lapse imaging in Fig 7D that were performed as in [51]. Briefly, pre-cultures of cells were grown over night in MSL+N at 25°C to reach an OD600 of between 0.5 and 1. Cultures were then diluted to an OD600 of 0.025 in MSL+N and grown for 18 hours to an OD600 of between 0.5 and 1 at 30°C. Cells were washed three times with MSL-N, diluted to an OD600 of 1.5 in 1 ml MSL-N and incubated at 30°C for 4 h. Cells were mounted onto MSL-N agarose pads (2% agarose) before imaging in overnight movies.

FRAP data in Figs 2, 3 and S2 were obtained with a Photokinesis module on a spinning disk confocal system previously described [35, 53]. The FRAP experiments described in Fig 2 were performed by bleaching a cortical region at the cell side, in S2 Fig by bleaching a cortical region that included half of the cell tip, in Fig 3 by bleaching the entire Gap1-GFP signal at the fusion site (in this case mating pairs with a stable fusion focus, visualized as a single Myo52-tdTomato dot, where selected, [35]). The selected region was bleached following two pre-bleach acquisitions and recovery was followed at regular intervals of 1”.

### Numerical method and simulation geometry

FRAP kinetics and reaction-diffusion patterns depend on a system’s dimensionality and geometry. We simulated these processes over a 3D curved surface representing the cell membrane. The simulated geometry was that of a cylinder with hemispherical caps at its two ends with radius 2 μm. The tip-to-tip cell length for simulations of mating cells was set to 6 μm and 10–12 μm for interphase cells, representative of experimental images. We simulated diffusion on a curved surface by implementing the algorithm of Novak et al. [54], in which the Laplace-Beltrami operator is approximated locally with the Laplacian operator over a tangential plane. The curved geometry of the cell membrane was discretized into a set of Voronoi polygons. The area of each Voronoi cell in the simulations was between 0.017 to 0.046 ${\mu m}^{2}$. We tested this algorithm by simulating diffusion with zero initial concentration except at a point placed on a cylinder or on spherical surface and comparing to the analytical solutions (for δ-function initial conditions) for these two cases.

We did not keep track of cytoplasmic concentrations, assuming that diffusion in the cytoplasm occurs with typical diffusion coefficients for single proteins. Such diffusion will smooth out cytoplasmic gradients over the Ras1-GTP patch size (~ 0.5 μm) over a time faster than the timescale of membrane binding and dissociation. Free diffusion across the cell through the cytoplasm would occur over seconds, which is much faster than the period of Ras1 patch appearance and disappearance (tens of seconds). Thus we also approximated uniform cytoplasmic concentration across the cell throughout the process of patch appearance and disappearance.

### Calculation of diffusion coefficients and rates of plasma membrane dissociation

For estimation of diffusion and rates of dissociation of Ras1-GDP and Ras1-GTP from the plasma membrane, we carried out FRAP experiments and fitted the data using numerical simulations.

To measure Ras1-GDP, we bleached GFP-Ras1 over a small (1.5±0.2μm) or a large (4.0±0.2μm) area at sides of a WT cells (Fig 2A). The acquired images of a single confocal slice through the bleached zone were corrected for photo bleaching by fitting an exponential decay function with decay constant $r_{PB}$ to the cytosolic signal after subtracting the out-of-cell background. The pixel intensity at every time point was corrected by multiplying by $1-e^{r_{PB}t}$ after subtracting the out-of-cell background. To quantify the recovery of GFP-Ras1 intensity over time, the average intensity of all the pixels within the bleached area was calculated and normalized with respect to the corresponding value at the pre-bleaching image. The normalized average intensity was calculated for 5 cells and their mean and standard deviation (Fig 2B). To measure Ras1-GTP, we performed the same analysis by bleaching GFP-Ras1 at the sides of constitutively active *ras1*^*Q66L*^ cells (Fig 2C and 2D).

We fitted the FRAP data with our 3D model, assuming uniform membrane diffusion and constant rates of association and dissociation to the membrane:

$$\frac{\partial C}{\partial t}=D\Delta_{S}C+j_{+}-rC$$ (1)

Here C is the concentration of Ras1-GDP or Ras1-GTP, *D* is the diffusion coefficient, $j_{+}$ is the association rate from the cytoplasm to the membrane and *r* is the dissociation rate from the membrane to the cytoplasm. The second order differential operator $\Delta_{S}$ is the Laplace–Beltrami operator. Starting from a system at steady state ($j_{+}=rC)$, bleaching is simulating by setting the concentration equal to zero for all Voronoi cells within a short (1.5μm) or long (4μm) region at the cell sides and along 6.28μm around the cylindrical circumference (inset of S1B and S1C Fig), using a time step 0.01 s.

By varying parameters D and r in Eq (1), we fitted the data in Fig 1B and 1D as well as the full recovery profiles (S1B–S1E Fig). To compare simulations to experimental data, we calculated the normalized concentrations on the Voronoi cells on a medial focal imaging plane and within 0.6μm in the vertical direction (approximate vertical width of the microscope’s point spread function). Diffusion results in different recovery rates between small and large regions, however small (large) D can be partly balanced by large (small) r (Fig 2E). The fits to the recovery curves of Fig 2B and 2D give $D_{RD}=0.03-0.15{\mu m}^{2}s^{-1},r_{RD}<0.03s^{-1},D_{RT}=0.02-0.07{\mu m}^{2}s^{-1},r_{RT}<0.025s^{-1}$ (Fig 2E). Further considering the fits to the full profiles (S1B–S1E Fig) provides a narrower range: $D_{RD}=0.145-0.155{\mu m}^{2}s^{-1},r_{RD}<0.005s^{-1},D_{RT}=0.035-0.045{\mu m}^{2}s^{-1},r_{RT}=0.018-0.022s^{-1}$.

For estimation of diffusion and dissociation rates of Gap1 a different approach compared to Ras1 had to be used because of its localized recruitment to the polarity patch. In this case we either bleached the full Gap1-GFP signal at the fusion focus in cells expressing Gap1-GFP and Myo52-tdTomato in mating conditions (Fig 3A) or we bleached half of Gap1-GFP at the cell tip during vegetative growth (S2A Fig). The acquired images were corrected for photobleaching prior to analysis as described above. For the Gap1-GFP full fusion focus recovery we observed a recovery up to 50%, which we interpret as a large portion of the cell’s Gap1 being bleached (Fig 3A and 3C). We identified the center and the side of the Gap1 distribution using the Myo52-tdTomato signal (Fig 3B). The average of intensity of all the pixels within each region shown in Fig 3B was calculated, normalized to the corresponding value at the pre-bleaching image. An example of the recovery at the middle region and side regions is shown in Fig 3C.

We used the 3D model to simulate the recovery of Gap1-GFP at the fusion focus using a model with localized recruitment of Gap1 to the cell tip (where the fusion focus is typically located), diffusion on the surface and uniform rate of membrane dissociation:

$$\frac{\partial C(r,t)}{\partial t}=D\Delta_{S}C(r,t)+\frac{A}{\sqrt{2\sigma^{2}\pi }}e^{-\frac{d^{2}(r)}{2\sigma^{2}}}-rC(r,t),$$ (2)

where C is the concentration of Gap1, *D* is the diffusion coefficient, r is the dissociation rate from the membrane to the cytoplasm, and d(r) is arc-length distance to the cell tip. The second term on right is the Gaussian recruitment function, with amplitude A and standard deviation σ=0.4μm estimated by measuring the full width at half maximum of Myo52-tdTomato signals at the fusion focus. The value of *A* was reduced to the half of the initial value after bleaching in the simulations to allow for 50% recovery similar to the experimental results (Figs 3C and S2E). A series of simulations starting with no Gap1 on the cell surface explored the dependence on the values of D and r. Then the average surface concentration of the Gap1 protein on the all Voronoi cells that were within the same middle and side area size as the experiments was calculated. This was compared to the normalized intensity of Gap1-GFP recovery at the middle and at the sides separately (Fig 3C). Good fits were obtained for $D=0.1-0.3{\mu m}^{2}s^{-1}$ and $r=0.015-0.025s^{-1}$.

The half-tip Gap1-GFP bleaching in the vegetative cells was done for the purpose of tracking diffusion from non-bleached region to the bleached region. In these experiments we observed a recovery up to 70% at the bleached region, which we interpret as a large portion of cellular Gap1 being bleached during these experiments (S2B and S2D Fig). We followed the changes in the intensity of Gap1-GFP in the bleached and non-bleached area separately after bleaching, as shown in S2B Fig. The average intensity of all the pixels within each region (~1.8 μm in width) was calculated and normalized to the corresponding value of the pre-bleaching image. The recovery of Gap1-GFP was averaged over 3 cells (S2D Fig) that had similar non-bleached area size, 2.8-3.1μm.

To model the half-tip Gap1-GFP recovery we used the same model discussed for full fusion focus with the difference in reducing the amplitude of Gaussian recruitment function after bleaching to 0.7 of initial value to account for the lost portion of Gap1-GFP by bleaching (S2C and S2D Fig). We let the system to reach steady state and then the Gap1 concentration was deleted over half of the tip as in the FRAP experiments. The standard deviation of the Gaussian function, σ, was estimated to be 0.8μm by comparing the full width at half maximum of Gap1-GFP in vegetative cells with the width at half maximum of Gap1-GFP at fusion focus in mating cells. The set of D, between $0.1{\mu m}^{2}s^{-1}$ to $0.3{\mu m}^{2}s^{-1}$, and $r=0.015-0.025s^{-1}$ values that were determined to be good fits from the full fusion focus Gap1-GFP recovery then were used for half-tip FRAP simulations to determine the best fit values $D=0.2{\mu m}^{2}s^{-1},r=0.02s^{-1}$ (S2D Fig). The curve with $D=0{\mu m}^{2}s^{-1}$ in S2D Fig is shown to demonstrate that cytoplasmic exchange by itself up to 70% of the initial amplitude cannot explain the loss of Gap1-GFP from the non-bleached region in the experiments even after adjusting r to the optimal value of $0.08s^{-1}$.

### Model of Ras1 exploratory zone and stabilization

The model is described by Eqs (3)–(7) presented in this section. The equations for Ras1-GTP and Ras1-GDP surface densities are as follows:

$$\frac{\partial C_{RT}}{\partial t}=D_{RT}\Delta_{S}C_{RT}+\left(k_{0}^{p}C_{GEF}+r_{noise}\right)C_{RD}-\left(k_{1}^{n}+k_{2}^{n}C_{GAP}\right)C_{RT}-r_{RT}C_{RT},$$ (3)

$$\frac{\partial C_{RD}}{\partial t}=D_{RD}\Delta_{S}C_{RD}+j_{RD}^{p}+\left(k_{1}^{n}+k_{2}^{n}C_{GAP}\right)C_{RT}-\left(k_{0}^{p}C_{GEF}+r_{noise}\right)C_{RD}-r_{RD}C_{RD}$$ (4)

where $\Delta_{S}$ is the Laplace–Beltrami diffusion operator, $k_{0}^{p}$is the GEF-mediated activation rate constant of Ras1-GDP, $k_{1}^{n}$ and $k_{2}^{n}$ are the rate constants of spontaneous and Gap1-mediated hydrolysis of Ras1-GTP, and $j_{RD}^{p}$is the uniform and constant rate of Ras1-GDP to the membrane from the cytoplasm. We use symbol r to label rate constants involving dissociation from membrane and k for rate constants involved in Ras1 activation or inactivation with superscript p or n, respectively. We also included a comparatively small random background activation of Ras1 by random variable $r_{noise}$, the magnitude of which was set to correspond to approximately 6 activated molecules per second per cell, the number used for Cdc42 in [16]. For more details see section “Implementation and effect of random Ras1 activation” below.

This model includes a positive feedback for activation of Ras1 by an autocatalytic interaction with GEFs. This is implemented similarly to the positive feedback in [16] by assuming GEFs are in quasi-static equilibrium with Ras1-GTP and assuming a quadratic non-linear dependence (the linear term was not sufficient for symmetry breaking in [16]):

$$C_{GEF}=k_{1}^{p}\frac{E_{c}}{V}C_{RT}+k_{2}^{p}\frac{E_{c}}{V}C_{RT}^{2}$$ (5)

Here $E_{c}$ is the available number of GEF molecules in the cytoplasm and *V* is the cell volume. The available number of GEF molecules in the cytoplasm in each time step, $E_{c}$, is calculated by $E_{c}={E_{c}}^{tot}-\int C_{GEF}da$, where ${E_{c}}^{tot}$ is the total number of GEF molecules in the cell and the integral is over the cell’s surface area, implying

$$E_{c}=E_{c}{}^{\text{tot}}/(1+\int \left[k_{1}^{p}\frac{1}{V}C_{RT}+k_{2}^{p}\frac{1}{V}C_{RT}^{2}\right]da).$$ (6)

The finite pool of GEF limits the number of patches that can form in the system and amount of activated Ras1 in the patch. See section “GEF Quasi-static equilibrium approximation” below for a more detailed examination of our assumptions on GEF properties.

There is also a negative feedback loop in this model to account for the role of Gap1. We assumed that Gap1 is recruited to the membrane through Ras1-GTP as previously shown [28] and similar to the model of negative feedback for Cdc42 oscillations in budding yeast [21]. The equation for Gap1 is as follows:

$$\frac{\partial C_{GAP}}{\partial t}=D_{GAP}\Delta_{S}C_{GAP}+k_{3}^{n}\frac{C_{RT}^{h}}{C_{sat}^{h}+C_{RT}^{h}}-r_{GAP}C_{GAP}$$ (7)

The non-linear recruitment rate of the second term on the right hand side was chosen to represent cooperative Gap1 recruitment at small Ras1-GTP concentrations, reaching a plateau for Ras1-GTP concentration above $C_{sat}.$ We used a value *h* = 2 that was the smallest integer value sufficient to provide a Ras1 exploratory zone.

Eqs (3)–(7) together with the model parameter values in Table 1 provide the complete model. For initialization, we start the simulations by setting $C_{RD}=j_{RD}^{p}/r_{RD}$plus or minus small relative random fluctuations, initialize a relatively smaller random Ras1-GTP field and set $C_{GAP}=0$ along the Voronoi cells representing the cell membrane.

### Implementation and effect of random Ras1 activation

The noise term in Eq (4), which represents fluctuations in Ras1 activation, is needed for the generation of a few Ras1-GTP to get the positive feedback started. We calculated the number d*N* of activated Ras1 per integration time interval d*t* over a Voronoi cell of surface area d*a* to be d*N* = d*t $da{pr}_{noise}C_{RD}$*, where *p* is a random number picked from a uniform probability distribution between 0 and 1. The corresponding change in the surface density was added to the corresponding Voronoi cell. This implementation of multiplicative noise (proportional to the surface density of Ras1-GDP) is specific to the chosen integration time interval, which we kept constant.

To study the effect of noise amplitude, we ran simulations with different values of $r_{noise}$, keeping the rest of the model parameters as in Table 1. The results of these simulations are summarized S7 Fig, demonstrating the period of patch appearance and disappearance as a function of $r_{noise}$ (with 0.002 $s^{-1}$ the default value). There is a minimum threshold $r_{noise}$ = 0.0005 $s^{-1}$ to get appearance and disappearance: below this threshold there is not enough spontaneous activation to trigger new patch formation. As $r_{noise}$ is increased above the default value, patch appearance and disappearance becomes more irregular and sometimes more two or more patches form in the simulations with one patch growing while other one shrinks/disappears or two competing patches forming simultaneously. There is an upper limit $r_{noise}$ = 0.008 $s^{-1}$ above which random activation results in uniform activation along the cell surface.

### Assumptions on GEF quasi-steady approximation

Here we discuss in more detail the assumption of GEF quasi-steady state of Eq (5). The precise mechanism by which Set6 gets recruited to the membrane and by which it diffuses on the membrane is not yet known. One way of achieving the required non-linear positive feedback is for Ste6 to get recruited by single Ras1-GTP molecules and by aggregates with two Ras1-GTP (possibly involving a scaffold protein). To check our assumption for GEF to be in quasi-static equilibrium, we added GEF binding and dissociation reactions to obtain the following expanded model:

$$\frac{\partial C_{RT}}{\partial t}=D_{RT}\Delta_{S}C_{RT}+(k_{0}^{p}C_{GEF}+r_{noise})C_{RD}-(k_{1}^{n}+k_{2}^{n}C_{GAP})C_{RT}-r_{RT}C_{RT}$$ (8)

$$\frac{\partial C_{GEF}}{\partial t}=D_{GEF}\Delta_{S}C_{GEF}+\rho_{1}\frac{E_{C}}{V}C_{RT}+\rho_{2}\frac{E_{C}}{V}C_{RT}^{2}-r_{GEF}C_{GEF}$$ (9)

$$\frac{\partial C_{RD}}{\partial t}=D_{RD}\Delta_{S}C_{RD}+j_{RD}^{p}+\left(k_{1}^{n}+k_{2}^{n}C_{GAP}\right)C_{RT}-\left(k_{0}^{p}C_{GEF}+r_{noise}\right)C_{RD}-r_{RD}C_{RD}$$ (10)

$$\frac{\partial C_{GAP}}{\partial t}=D_{GAP}\Delta_{S}C_{GAP}+k_{3}^{n}\frac{C_{RT}^{h}}{C_{sat}^{h}+C_{RT}^{h}}-r_{GAP}C_{GAP}$$ (11)

$$E_{c}=E_{c}^{tot}-\int C_{GEF}da$$ (12)

The new equation for $C_{GEF}$ describes the surface density of Ste6, $\rho_{1}$ is the rate constant for recruitment of Ste6 to the membrane by single Ras1-GTP molecules and $\rho_{2}$ the rate constant of Ste6 recruitment by aggregates with two Ras1-GTP. We use the same membrane diffusion coefficient for membrane bound GEF as for Ras1-GTP. To choose $\rho_{1}$ and $\rho_{2}$ parameters consistent with the model of Eqs (3)–(7), we relate them to parameters $k_{1}^{p}$ and $k_{2}^{p}$ via:

$$k_{1}^{p}=\rho_{1}/r_{GEF}$$ (13)

$$k_{2}^{p}=\rho_{2}/r_{GEF}$$ (14)

In the limit of fast GEF dissociation (large $r_{GEF}$), the reaction terms on the right hand side of Eq (9) balance, resulting in Eq (5).

We found that using $r_{GEF}$ = 10 $s^{-1}$ and other parameters as in Table 1 (except for the need to use a smaller integration time step of 10^−5^ s) produced patch appearance and disappearance similar to Fig 5, with a slightly shorter period of 45 sec. For $r_{GEF}=0.1,1s^{-1}$ the patch did not reform after an initial appearance and disappearance. Thus the model of Eqs (3)–(7) requires that the patch establishes biochemical equilibrium faster than a sec timescale.

The requirement for $r_{GEF}$ ~ 10 $s^{-1}$is of the same order as the value of 10 $s^{-1}$ for Cdc24 GEF dissociation from the budding yeast Cdc42 patch in the model of Goryachev and Pokhilko [16]. However we note that these authors used a different GEF mechanism compared to Eqs (8)–(12). In their study, positive Cdc42 feedback arises from the combination of slow uniform GEF recruitment to the membrane (similar to random Ras1 activation in our model, needed to get the Ras1 activation started) followed by GEF-mediated Cdc42 activation and then complex formation of GEF with active Cdc42.

Finally, we note that Ste6 transcription is a target of the MAPK cascade and so it may take a while after pheromone exposure until it reaches steady state. However, patch dynamics continue in much the same way for at least 14h in cells without partners, when Ste6 levels should be at steady state. Thus, modulations in Ste6 expression levels are not likely to play a role in patch dynamics.

### Estimation of Ras1-GTP hydrolysis rate

To calculate the spontaneous Ras1-GTP hydrolysis rate, we fitted the in vitro Ras1-GTP hydrolysis data of (Fig 4A in [23]) with an exponential decay function. This leads to a spontaneous hydrolysis rate $k_{1}^{n}=(1.23\pm 0.05)\times 10^{-3}s^{-1}$. To estimate the Gap1-dependent hydrolysis rate we fitted the GST-Ras1 + MPB Gap1-1 graph (Fig 4A in [23]) with an exponential, which gives a decay rate $0.00293s^{-1}$. Assuming that this rate is linear in Gap1 concentration and equal to $k_{1}^{n}+k_{2}^{n,3D}C_{GAP}^{3D}$, where 3D indicated concentrations per unit volume (as opposed to per unit area) we obtain $k_{2}^{n,3D}=7.2\times 10^{-7}{\mu m}^{3}s^{-1}$. Assuming that Gap1 bound to the cell membrane is within *w* = 10 nm off the cell membrane gives an estimate for the Gap1 dependent hydrolysis rate for the model $k_{2}^{n}=k_{2}^{n,3D}/w=7.2\times 10^{-5}{\mu m}^{2}s^{-1}$. In the model we had to use a larger value (0.1 ${\mu m}^{2}s^{-1})$ such that a realistic concentration of Gap1 at the membrane is sufficient to cause zone disassembly. The latter is not an unlikely possibility since Gap1 may be better positioned for hydrolysis when bound to the membrane in cells. Use of a higher Ras1 spontaneous hydrolysis rate in the model does not change the previous conclusions though it brings the system closer to the winner-take-all mechanism, with less role of Gap1 in determining the zone size.

### Quantification of patch size and frequency of simultaneous number of patches

Wt, Ste6 overexpression and Gap1 overexpression cells expressing RasAct^GFP^ and Myo52-tdTomato, imaged every 10 minutes on a DeltaVision platform, showed exploratory patch of RasAct^GFP^ that appeared at different position along the cell cortex in early stages of mating. We identified any bright region on the cell cortex which was equal or larger than two pixels and was brighter than the average cell background signal. By drawing a line manually along the bright regions their size and average intensity was measured. We also measured the cell background and standard deviation between the intensity of cell background pixels by drawing an irregular shape inside the cell excluding the nucleus. RasAct^GFP^ patch was defined as any bright region with an average intensity higher than 3 times of standard deviation between the intensity of cell background pixels. The cytoplasmic intensity and its standard deviation was similar in all three cases (S6 Fig). Since RasAct^GFP^ has a spottier distribution compared to other patch markers such as Scd2, RasAct^GFP^ regions that were close to one another (of order 2 pixels = 260 nm) were counted as a single patch. We also quantified the number of simultaneous patches along the cell cortex in each cell type. Then we averaged over the patch sizes in every cell type and performing two sample t-test between the patch size mean of wt and Ste6/Gap1 overexpression mutants we could show that the averages are significantly different, P-value < 0.001.

## Supporting information

S1 Software Source code and jar executable of model is available as supporting information. The code uses the Open Source Physics java library.
(ZIP)

S1 Movie Simulation results with parameters values from Table 1 showing appearance and disappearance of an exploratory patch. Image shows two sides and two panels for concentration of Ras1-GTP/Ras1-GDP (red/blue) and Gap1 (green) of a single simulated cell.
(MOV)

S2 MovieSimulations showing formation and competition of two patches within the appearance and disappearance time period as a result of increase in the the positive feedback rate constant $k_{0}^{p}$ by 1.6 times.(MOV)

S1 FigFit of Ras1 FRAP profile along cell side.(A) Localization of GFP-Ras1 in wt and RasAct^GFP^, a reporter for Ras1-GTP, in wt and *ras1*^*Q66L*^ cells during vegetative growth. The scale bar is 2 μm. (B) Normalized intensity profile of GFP-Ras1 recovery at the sides of a WT cell in FRAP experiment of cell in Fig 2A. Smooth lines show the corresponding fitted curves by a model with *D* = 0.15 ${\mu m}^{2}s^{-1}$ and no cytoplasmic exchange. Inset shows snapshots of simulation. (C) Similar to panel A for a smaller bleached region and same model parameters. (D) Normalized intensity profile of GFP-Ras1 recovery at the sides of the *ras1*^*Q66L*^ cell shown in Fig 2C. Smooth lines show the corresponding fitted curves by a model with *D* = 0.04 ${\mu m}^{2}s^{-1}$, and uniform cytoplasmic exchange rate 0.02 $s^{-1}$. (E) Similar to panel C for a smaller bleached region and same model parameters.(EPS)

S2 FigHalf-tip bleach of Gap1 and model fit.(A) Snapshots of FRAP of Gap1-GFP after bleaching half of a WT cell tip (red star). The scale bar is 1 μm. (B) Intensity profile along the tip at the indicated time points for cell in panel A. Blue (red) double arrow shows a segment of the non-bleached (bleached) region. (C) Intensity profile along the tip at the indicated time points from simulations of a model with a Gaussian function for recruitment of Gap1-GFP to the cell tip, *D* = 0.2 ${\mu m}^{2}s^{-1}$, and uniform cytoplasmic exchange rate 0.2 $s^{-1}$. (D) Recovery of Gap1-GFP at the bleached region and decay of Gap1-GFP at the non-bleached region indicated in panel B, average of 3 cells. Continuous curves show fits by model with a recruitment of Gap1-GFP to the cell tip with the indicated values of *D* and uniform cytoplasmic exchange rate, assuming 70% of Gap1-GFP in the cell is photobleached. (E) Intensity profile along the cell tip over time from simulations with a Gaussian function for recruitment of Gap1-GFP to the cell tip, *D* = 0.2 ${\mu m}^{2}s^{-1}$, and cytoplasmic exchange rate 0.02 $s^{-1}$.(EPS)

S3 Fig Simulations showing evolution of Ras1 patch formation and disappearance over time. (A) Surface density profile of Ras1-GTP over a 0.2 μm wide line along the long axis of the cell and through the center of the patch at the indicated time points for the simulation shown in Fig 4B. (B) Same as panel A, for Gap1. (C) Same as panel A, for Ras1-GDP. (D) Same as panel A, for GEF.
(EPS)

S4 FigDynamical behavior in different regions of parameter space.Behavior of simulations behavior for different values of $C_{sat}$, the saturation parameter in Gap1 recruitment term, and $k_{2}^{n}$, the Gap1-dependent hydrolysis rate constant of Ras1-GTP. Other free parameters were kept constant as shown in Table 1. The yellow region represent simulations that mostly show a single patch oscillating however sometimes there are two patches that form simultaneously and then disappear at the same time or one after the other. One pixel* is one voronoi cell on the simulated cell surface.(EPS)

S5 FigSimulations of Ras1 patch size regulation through positive and negative feedback.(A) Similar to Fig 7A, surface density profile over a 0.2 μm wide strip along the cell long axis going through the center of an exploring patch for simulations with stronger negative feedback compared to Table 1. Solid lines: increase of Gap1-dependent hydrolysis of Ras1-GTP rate constant $k_{2}^{n}$by 10 times. Dotted lines: reference curves from Fig 4D. (B) Similar to Fig 7B, surface density profile over a 0.2 μm wide strip along the cell long axis going through the center of two exploring aligned patches for stronger positive feedback rate constants (10-fold increase of $k_{1}^{p}$ and $k_{2}^{p}$ across cell surface). (C) similar to Fig 7C, surface density profile over a 0.2 μm wide strip along the cell long axis going through the center of a patch stabilized via stronger local positive feedback. Curves show effect of change with respect to values of Table 1: (i) increase of Ras1 activation rate constant $k_{0}^{p}$by 1.2 times; (ii) increase of $k_{0}^{p}$by 1.2 times and increase of available GEF molecules in the cell by 1.1 times. (D) Simulation snapshots showing examples of simultaneous formation of two exploring patches as a result of increasing the total amount of GEF molecules in the system, $E_{c}^{tot}$, by 1.5 while keeping the rest of the parameters as in Table 1.(EPS)

S6 Fig RasAct^GFP^ patch intensity analysis in WT, Ste6 overexpression and Gap1 overexpression cells. (A) RasAct^GFP^ patch average intensity over cytoplasmic background was measured in WT (black, *n* = 404 patches in 23 cells), Ste6 overexpression (red, *n* = 467 patches in 28 cells) and Gap1 overexpression (*n* = 219 patches in 24 cells) cells expressing RasAct^GFP^ (in blue) and Myo52-tdTomato. (B) Average cytoplasmic background for the patch intensity measurements in panel A. (C) The average standard deviation within the cytoplasmic background for the patch intensity measurements in panel A. Gray lines in all panels show standard error.
(EPS)

S7 FigEffect of Ras1 activation noise amplitude in patch period.Patch appearance and disappearance period for different values of $r_{noise}$, keeping the rest of the model parameters same as in Table 1. The reference value is $r_{noise}$ = 0.002 $s^{-1}.$The patch is stable below $r_{noise}$ = 0.0005 $s^{-1}$. As $r_{noise}$ is increased above the default value, patch appearance and disappearance becomes more irregular and sometimes more two or more patches form in the simulations with one patch growing while other one shrinks/disappears or two competing patches forming simultaneously. Above $r_{noise}$ = 0.008 $s^{-1}$ no patch is formed for a system initialized with no activated Ras1 at *t* = 0.(EPS)

## References

[1] Levchenko A, Iglesias PA. Models of Eukaryotic Gradient Sensing: Application to Chemotaxis of Amoebae and Neutrophils. Biophysical Journal. 2002;82(1):50–63. 11751295 10.1016/S0006-3495(02)75373-3 PMC1302448

[2] Schneider IC, Haugh JM. Mechanisms of Gradient Sensing and Chemotaxis Conserved Pathways, Diverse Regulation. Cell Cycle. 2006;5(11):1130–4. 10.4161/cc.5.11.2770 16760661

[3] Arkowitz RA. Chemical Gradients and Chemotropism in Yeast. Cold Spring Harbor perspectives in biology. 2009;1(2). 10.1101/cshperspect.a001958 PMC2742094 20066086

[4] Devreotes P, Horwitz AR. Signaling networks that regulate cell migration. Cold Spring Harbor perspectives in biology. 2015;7(8):a005959 10.1101/cshperspect.a005959 26238352 PMC4526752

[5] Park H-O, Bi E. Central Roles of Small GTPases in the Development of Cell Polarity in Yeast and Beyond. Microbiology and Molecular Biology Reviews. 2007;71(1):48–96. 10.1128/MMBR.00028-06 17347519 PMC1847380

[6] Bardwell L. A walk-through of the yeast mating pheromone response pathway. Peptides. 2005;26(2):339–50. 15690603 10.1016/j.peptides.2004.10.002 PMC3017506

[7] Merlini L, Dudin O, Martin SG. Mate and fuse: how yeast cells do it. Open biology. 2013;3(3):130008 10.1098/rsob.130008 23466674 PMC3718343

[8] Martin SG, Arkowitz RA. Cell polarization in budding and fission yeasts. FEMS Microbiology Reviews. 2014;38(2):228–53. 10.1111/1574-6976.12055 24354645

[9] Bendezú FO, Martin SG. Cdc42 Explores the Cell Periphery for Mate Selection in Fission Yeast. Current biology: CB. 2013;23(1):42–7. 10.1016/j.cub.2012.10.042 23200991

[10] Hegemann B, Unger M, Lee SS, Stoffel-Studer I, van den Heuvel J, Pelet S, et al A Cellular System for Spatial Signal Decoding in Chemical Gradients. Developmentall Cell. 2015;35:458–70. 10.1016/j.devcel.2015.10.013 26585298

[11] Merlini L, Khalili B, Bendezú FO, Hurwitz D, Vincenzetti V, Vavylonis D, et al Local Pheromone Release from Dynamic Polarity Sites Underlies Cell-Cell Pairing during Yeast Mating. Current biology: CB. 2016;26(8):1117–25. 10.1016/j.cub.2016.02.064 27020743 PMC4846541

[12] Dyer Jayme M, Savage Natasha S, Jin M, Zyla Trevin R, Elston Timothy C, Lew Daniel J. Tracking Shallow Chemical Gradients by Actin-Driven Wandering of the Polarization Site. Current Biology. 2013;23(1):32–41. 10.1016/j.cub.2012.11.014 23200992 PMC3543483

[13] McClure Allison W, Minakova M, Dyer Jayme M, Zyla Trevin R, Elston Timothy C, Lew Daniel J. Role of Polarized G Protein Signaling in Tracking Pheromone Gradients. Developmental Cell. 2015;35(4):471–82. 10.1016/j.devcel.2015.10.024 26609960 PMC4661441

[14] Slaughter BD, Smith SE, Li R. Symmetry Breaking in the Life Cycle of the Budding Yeast. Cold Spring Harbor perspectives in biology. 2009;1(3). 10.1101/cshperspect.a003384 PMC2773630 20066112

[15] Meinhardt H. Orientation of chemotactic cells and growth cones: models and mechanisms. J Cell Sci. 1999;112 (Pt 17):2867–74. 10444381 10.1242/jcs.112.17.2867

[16] Goryachev AB, Pokhilko AV. Dynamics of Cdc42 network embodies a Turing-type mechanism of yeast cell polarity. FEBS letter. 2008;582(10):1437–43. 10.1016/j.febslet.2008.03.029 18381072

[17] Savage NS, Layton AT, Lew DJ. Mechanistic mathematical model of polarity in yeast. Molecular Biology of the Cell. 2012;23(10):1998–2013. 10.1091/mbc.E11-10-0837 22438587 PMC3350562

[18] Freisinger T, Klunder B, Johnson J, Muller N, Pichler G, Beck G, et al Establishment of a robust single axis of cell polarity by coupling multiple positive feedback loops. Nat Commun. 2013;4:1807 10.1038/ncomms2795 23651995 PMC3674238

[19] Martin SG. Spontaneous cell polarization: Feedback control of Cdc42 GTPase breaks cellular symmetry. Bioessays. 2015;37(11):1193–201. 10.1002/bies.201500077 26338468

[20] Woods B, Lai H, Wu C-F, Zyla Trevin R, Savage Natasha S, Lew Daniel J. Parallel Actin-Independent Recycling Pathways Polarize Cdc42 in Budding Yeast. Current Biology. 2016;26(16):2114–26. 10.1016/j.cub.2016.06.047 27476596 PMC5956535

[21] Howell AS, Jin M, Wu CF, Zyla TR, Elston TC, Lew DJ. Negative feedback enhances robustness in the yeast polarity establishment circuit. Cell. 2012;149(2):322–33. 10.1016/j.cell.2012.03.012 22500799 PMC3680131

[22] Nadin-Davis SA, Nasim A, Beach D. Involvement of ras in sexual differentiation but not in growth control in fission yeast. The EMBO journal. 1986;5(11):2963–71. 16453723 10.1002/j.1460-2075.1986.tb04593.x PMC1167248

[23] Merlini L, Khalili B, Dudin O, Vincenzetti V, Martin SG. Inhibition of Ras activity coordinates cell fusion with cell-cell contact during yeast mating. Journal of Cell Biology. 2018;217:1467–83. 10.1083/jcb.201708195 29453312 PMC5881505

[24] Papadaki P, Pizon V, Onken B, Chang EC. Two ras pathways in fission yeast are differentially regulated by two ras guanine nucleotide exchange factors. Mol Cell Biol. 2002;22(13):4598–606. 10.1128/MCB.22.13.4598-4606.2002 12052869 PMC133927

[25] Hughes DA, Yabana N, Yamamoto M. Transcriptional regulation of a Ras nucleotide-exchange factor gene by extracellular signals in fission yeast. J Cell Sci. 1994;107 (Pt 12):3635–42. 7706412 10.1242/jcs.107.12.3635

[26] Mata J, Bahler J. Global roles of Ste11p, cell type, and pheromone in the control of gene expression during early sexual differentiation in fission yeast. Proceedings of the National Academy of Sciences of the United States of America. 2006;103(42):15517–22. 10.1073/pnas.0603403103 17032641 PMC1592531

[27] Das M, Drake T, Wiley DJ, Buchwald P, Vavylonis D, Verde F. Oscillatory Dynamics of Cdc42 GTPase in the Control of Polarized Growth. Science. 2012 337:239–243. 10.1126/science.1218377 22604726 PMC3681419

[28] Weston C, Bond M, Croft W, Ladds G. The coordination of cell growth during fission yeast mating requires Ras1-GTP hydrolysis. PloS one. 2013;8(10):e77487 10.1371/journal.pone.0077487 24147005 PMC3797800

[29] Marguerat S, Schmidt A, Codlin S, Chen W, Aebersold R, Bahler J. Quantitative analysis of fission yeast transcriptomes and proteomes in proliferating and quiescent cells. Cell. 2012;151(3):671–83. 10.1016/j.cell.2012.09.019 23101633 PMC3482660

[30] Dudin O, Merlini L, Martin SG. Spatial focalization of pheromone/MAPK signaling triggers commitment to cell-cell fusion. Genes & development. 2016;30(19):2226–39. 27798845 10.1101/gad.286922.116 PMC5088570

[31] Chiou J-g, Balasubramanian MK, Lew DJ. Cell Polarity in Yeast. Annual Review of Cell and Developmental Biology. 2017;33(1):77–101. 10.1146/annurev-cellbio-100616-060856 PMC5944360 28783960

[32] Xiong Y, Huang C-H, Iglesias PA, Devreotes PN. Cells navigate with a local-excitation, global-inhibition-biased excitable network. Proceedings of the National Academy of Sciences. 2010;107(40):17079–86. 10.1073/pnas.1011271107 PMC2951443 20864631

[33] Howell AS, Savage NS, Johnson SA, Bose I, Wagner AW, Zyla TR, et al Singularity in polarization: re-wiring yeast cells to make two buds. Cell. 2009;139(4):731–43. 10.1016/j.cell.2009.10.024 19914166 PMC2783644

[34] Chang EC, Barr M, Wang Y, Jung V, Xu HP, Wigler MH. Cooperative interaction of S. pombe proteins required for mating and morphogenesis. Cell. 1994;79(1):131–41. 7923372 10.1016/0092-8674(94)90406-5

[35] Dudin O, Bendezú FO, Groux R, Laroche T, Seitz A, Martin SG. A formin-nucleated actin aster concentrates cell wall hydrolases for cell fusion in fission yeast. J Cell Biol. 2015;208(7):897–911. 10.1083/jcb.201411124 25825517 PMC4384723

[36] Bonazzi D, Haupt A, Tanimoto H, Delacour D, Salort D, Minc N. Actin-Based Transport Adapts Polarity Domain Size to Local Cellular Curvature. Current biology: CB. 2015;25(20):2677–83. 10.1016/j.cub.2015.08.046 26441355

[37] Bendezú FO, Vincenzetti V, Vavylonis D, Wyss R, Vogel H, Martin SG. Spontaneous Cdc42 Polarization Independent of GDI-Mediated Extraction and Actin-Based Trafficking. PloS Biol. 2015;13(4): e1002097 10.1371/journal.pbio.1002097 25837586 PMC4383620

[38] Vinnakota KC, Mitchell DA, Deschenes RJ, Wakatsuki T, Beard DA. Analysis of Diffusion of Ras2 in Saccharomyces cerevisiae Using Fluorescence Recovery after Photobleaching. Physical biology. 2010;7(2):026011 10.1088/1478-3975/7/2/026011 20526029 PMC2897723

[39] Niv H, Gutman O, Henis YI, Kloog Y. Membrane Interactions of a Constitutively Active GFP-Ki-Ras 4B and Their Role in Signaling: EVIDENCE FROM LATERAL MOBILITY STUDIES. Journal of Biological Chemistry. 1999;274(3):1606–13. 9880539 10.1074/jbc.274.3.1606

[40] Lommerse PHM, Snaar-Jagalska BE, Spaink HP, Schmidt T. Single-molecule diffusion measurements of H-Ras at the plasma membrane of live cells reveal microdomain localization upon activation. Journal of Cell Science. 2005;118(9):1799. 15860728 10.1242/jcs.02300

[41] Niv H, Gutman O, Kloog Y, Henis YI. Activated K-Ras and H-Ras display different interactions with saturable nonraft sites at the surface of live cells. The Journal of Cell Biology. 2002;157(5):865 10.1083/jcb.200202009 12021258 PMC2173426

[42] Prior IA, Hancock JF. Ras trafficking, localization and compartmentalized signalling. Seminars in Cell & Developmental Biology. 2012;23(2):145–53. 21924373 10.1016/j.semcdb.2011.09.002 PMC3378476

[43] Zhou Y, Hancock JF. Ras nanoclusters: Versatile lipid-based signaling platforms. Biochimica et Biophysica Acta (BBA)—Molecular Cell Research. 2015;1853(4):841–9. 25234412 10.1016/j.bbamcr.2014.09.008

[44] Andrew N, Insall RH. Chemotaxis in shallow gradients is mediated independently of PtdIns 3-kinase by biased choices between random protrusions. Nat Cell Biol. 2007;9(2):193–200. 10.1038/ncb1536 17220879

[45] Jilkine A, Edelstein-Keshet L. A Comparison of Mathematical Models for Polarization of Single Eukaryotic Cells in Response to Guided Cues. PLOS Computational Biology. 2011;7(4):e1001121 10.1371/journal.pcbi.1001121 21552548 PMC3084230

[46] Larsch J, Flavell SW, Liu Q, Gordus A, Albrecht DR, Bargmann CI. A circuit for gradient climbing in C. elegans chemotaxis. Cell reports. 2015;12(11):1748–60. 10.1016/j.celrep.2015.08.032 26365196 PMC5045890

[47] Jilkine A, Angenent SB, Wu LF, Altschuler SJ. A Density-Dependent Switch Drives Stochastic Clustering and Polarization of Signaling Molecules. PLOS Computational Biology. 2011;7(11):e1002271 10.1371/journal.pcbi.1002271 22102805 PMC3213192

[48] Insall RH. Understanding eukaryotic chemotaxis: a pseudopod-centred view. Nat Rev Mol Cell Biol. 2010;11(6):453–8. 10.1038/nrm2905 20445546

[49] Takeda K, Shao D, Adler M, Charest PG, Loomis WF, Levine H, et al Incoherent Feedforward Control Governs Adaptation of Activated Ras in a Eukaryotic Chemotaxis Pathway. Science Signaling. 2012;5(205):ra2 10.1126/scisignal.2002413 22215733 PMC3928814

[50] Egel R, Willer M, Kjaerulff S, Davey J, Nielsen O. Assessment of pheromone production and response in fission yeast by a halo test of induced sporulation. Yeast. 1994;10(10):1347–54. 10.1002/yea.320101012 7900424

[51] Vjestica A, Merlini L, Dudin O, Bendezu FO, Martin SG. Microscopy of Fission Yeast Sexual Lifecycle. J Vis Exp. 2016 10.3791/53801 27022830 PMC4828234

[52] Bähler J, Wu JQ, Longtine MS, Shah NG, McKenzie A 3rd, Steever AB, et al Heterologous modules for efficient and versatile PCR-based gene targeting in Schizosaccharomyces pombe. Yeast. 1998;14(10):943–51. 10.1002/(SICI)1097-0061(199807)14:10<943::AID-YEA292>3.0.CO;2-Y 9717240

[53] Bendezú FO, Martin SG. Actin cables and the exocyst form two independent morphogenesis pathways in the fission yeast. Mol Biol Cell. 2011;22(1):44–53. 10.1091/mbc.E10-08-0720 21148300 PMC3016976

[54] Novak IL, Gao F, Choi Y, Resasco D, Schaff JC, Slepchenko BM. Diffusion on a curved surface coupled to diffusion in the volume: Application to cell biology. Journal of Computational Physics. 2007;226(2):1271–90. 10.1016/j.jcp.2007.05.025 18836520 PMC2346449

